# The NLRP3 inflammasome is involved with the pathogenesis of Mayaro virus

**DOI:** 10.1371/journal.ppat.1007934

**Published:** 2019-09-03

**Authors:** Luiza A. de Castro-Jorge, Renan V. H. de Carvalho, Taline M. Klein, Carlos H. Hiroki, Alexandre H. Lopes, Rafaela M. Guimarães, Marcílio Jorge Fumagalli, Vitor G. Floriano, Mayara R. Agostinho, Renata Dezengrini Slhessarenko, Fernando Silva Ramalho, Thiago M. Cunha, Fernando Q. Cunha, Benedito A. L. da Fonseca, Dario S. Zamboni

**Affiliations:** 1 Department of Cell Biology, School of Medicine of Ribeirão Preto, University of São Paulo. Ribeirão Preto, Brazil; 2 Department of Internal Medicine, School of Medicine of Ribeirão Preto, University of São Paulo. Ribeirão Preto, Brazil; 3 Center for Research in Inflammatory Diseases, Department of Pharmacology, School of Medicine of Ribeirão Preto, University of São Paulo. Ribeirão Preto, Brazil; 4 School of Medicine, Federal University of Mato Grosso, Cuiabá, Brazil; 5 Department of Pathology, School of Medicine of Ribeirão Preto, University of São Paulo. Ribeirão Preto, Brazil; The University of Chicago, UNITED STATES

## Abstract

Mayaro virus (MAYV) is an arbovirus that circulates in Latin America and is emerging as a potential threat to public health. Infected individuals develop Mayaro fever, a severe inflammatory disease characterized by high fever, rash, arthralgia, myalgia and headache. The disease is often associated with a prolonged arthralgia mediated by a chronic inflammation that can last months. Although the immune response against other arboviruses, such as chikungunya virus (CHIKV), dengue virus (DENV) and Zika virus (ZIKV), has been extensively studied, little is known about the pathogenesis of MAYV infection. In this study, we established models of MAYV infection in macrophages and in mice and found that MAYV can replicate in bone marrow-derived macrophages and robustly induce expression of inflammasome proteins, such as NLRP3, ASC, AIM2, and Caspase-1 (CASP1). Infection performed in macrophages derived from *Nlrp3*^*–/–*^, *Aim2*^*–/–*^, *Asc*^*–/–*^and *Casp1/11*^*–/–*^mice indicate that the NLRP3, but not AIM2 inflammasome is essential for production of inflammatory cytokines, such as IL-1β. We also determined that MAYV triggers NLRP3 inflammasome activation by inducing reactive oxygen species (ROS) and potassium efflux. *In vivo* infections performed in inflammasome-deficient mice indicate that NLRP3 is involved with footpad swelling, inflammation and pain, establishing a role of the NLRP3 inflammasome in the MAYV pathogenesis. Accordingly, we detected higher levels of caspase1-p20, IL-1β and IL-18 in the serum of MAYV-infected patients as compared to healthy individuals, supporting the participation of the NLRP3-inflammasome during MAYV infection in humans.

## Author summary

Viruses transmitted by mosquitoes have recently received huge attention from the media because the epidemics caused by Zika and chikungunya virus rapidly spread to new areas and infected a large number of individuals. Mayaro is a virus transmitted by mosquitoes that circulates in the Caribbean and tropical regions of Latin America. It causes a highly inflammatory disease, called Mayaro fever, and acute disease is often followed by a prolonged arthralgia mediated by chronic inflammation that can last months or years. The spread of Mayaro to urban areas is a major concern by the authorities, given that the virus has potential to cause an epidemic if spread in high-risk areas. Thus, understanding the mechanisms related to the pathogenesis of this infectious agent would be of great value to treat and prevent the disease. Here, we established an adult mouse model of Mayaro infection and demonstrated that the virus activates the NLRP3 inflammasome, which is important to regulate this viral disease. Our study provides molecular targets for a future treatment of Mayaro fever and provides an experimental model to understand the pathology caused by this emerging viral pathogen.

## Introduction

Arboviruses are one of the public health authorities major concerns, contributing to an increasing awareness of emerging infections worldwide. After the spread of chikungunya virus (CHIKV) to new areas of the globe and the emergence of Zika virus (ZIKV), surveillance systems worldwide are focusing much attention on tracking the next arboviral epidemic [1]. Reported human cases of Mayaro virus (MAYV) infection have been limited to Central and South America, particularly to the regions around the Amazon basin [2–5]. Recent studies revealed the emergence of MAYV recombinants in Brazil and Haiti, and adaptation to a broad host and vector range, placing these countries as high-risk areas for the emergence of MAYV epidemics [6,7].

MAYV belongs to the *Togaviridae* family and *Alphavirus* genus, which consists of well-known pathogenic viruses, such as CHIKV, Ross River virus (RRV), Eastern (EEEV), Western (WEEV), and Venezuelan equine encephalitis (VEEV) viruses [8]. *Haemagogus* mosquitoes have been documented as the main vectors of MAYV, and *Aedes aegypti* has also been found to be a competent vector, a feature that has alerted authorities to the eminent possibility that MAYV emerge as a global pathogen [1,9]. MAYV is the causative agent of Mayaro fever, a neglected endemic infection that is characterized by nonspecific symptoms such as high fever, rash, arthralgia, myalgia and headache. Similar to CHIKV infection, MAYV is associated with a prolonged arthralgia which can last for months or even years, possibly due to chronic inflammation [10,11]. However, the mechanisms underlying these clinical signs are still not elucidated.

Inflammation is a key event during the pathogenesis of many diseases [12–15], and is also the case of those caused by arbovirus [11]. Several studies have characterized the inflammatory process of dengue virus (DENV), CHIKV and ZIKV infection, both in animal models and humans [16–18]. For example, the acute phase of CHIKV infection is associated with high production of inflammatory mediators including IL-6, IL-8, IL-12 and MCP-1, while the chronic phase is associated with other inflammatory cytokines, such as IL-17, IFN-γ and IL-1β [11]. Overall, these cytokines are mainly produced upon recognition of pathogens by pattern recognition receptors (PRRs), such as Toll-like receptors (TLRs) and Nod-like receptors (NLRs) [19,20]. While TLRs are found within cellular and endosomal membranes, NLRs are located in the cytoplasm. They can be activated by different types of pathogens and their associated molecular patterns (PAMPs) [21,22], or by generation of damage-associated molecular patterns (DAMPs) such as potassium efflux, reactive oxygen species (ROS) production, and cathepsin B release [12,23].

Upon activation, NLRs trigger the assembly of cytosolic protein complexes called inflammasomes, which consists of a NLR protein, an adaptor protein, and caspase-1. This enzyme is capable of cleaving pro-IL-1β, producing its mature form (IL-1β), and inducing an inflammatory type of cell death called pyroptosis [23,24]. Although several inflammasomes have been described, the NLRP3 inflammasome is the most studied. It is involved in a wide variety of diseases, such as autoimmunity, cancer and neurodegenerative and infectious diseases [12,23,25]. Arboviral infections, including ZIKV and CHIKV can trigger NLRP3 inflammasome activation in myeloid cells, such as macrophages, leading to an increased production of IL-1β, contributing to pathological inflammatory events that drive the development of both diseases [16,17]. However, whether MAYV infection leads to activation of NLRs and inflammasome assembly has never been reported *in vitro* or *in vivo*.

MAYV-induced inflammation is likely to play a key role during MAYV pathogenesis, as suggested by a study conducted in patients [11]. Although some studies have evaluated MAYV infection in different models of mice [26,27], the mechanisms governing the pathogenesis of the disease remain largely unexplored. Here, we used primary bone marrow-derived macrophages (BMDMs) to investigate infection and replication of MAYV, we also established an adult mouse model of acute inflammation that allows the evaluation of effects of inflammasomes during the pathogenesis of MAYV. Our study highlights the key role of the NLRP3 inflammasome for pathogenesis of MAYV infection.

## Results

### MAYV infection triggers robust inflammasome activation in BMDMs

**Fig 1:**
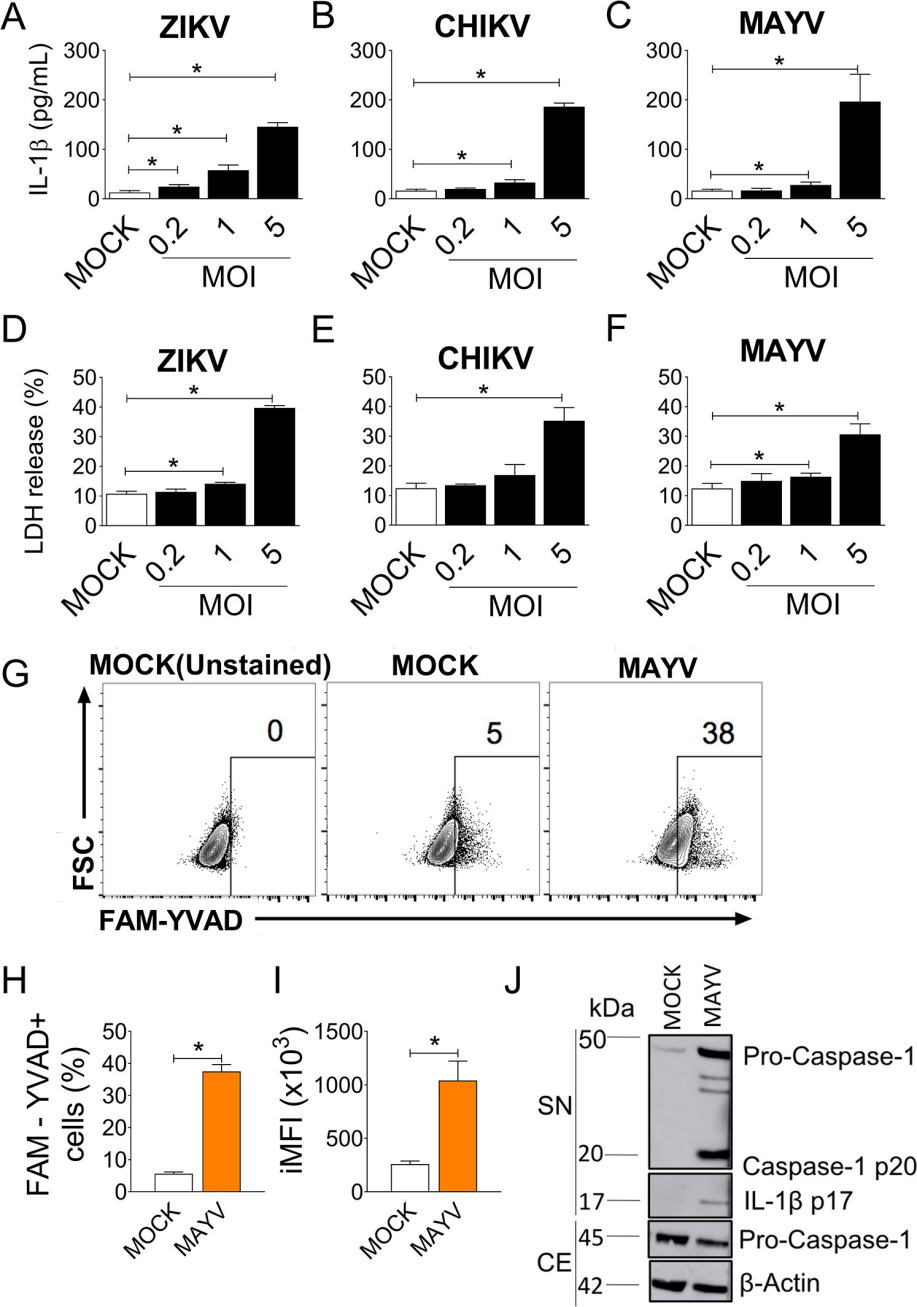
MAYV infection trigger caspase-1 activation in macrophages. (A-F) PAM(3)CSK(4)-primed bone marrow-derived macrophages (BMDMs) were treated with mock or infected with Zika virus (ZIKV), chikungunya virus (CHIKV) or Mayaro virus (MAYV) at a MOI of 0.2, 1 or 5. After 24 hours of infection, the levels of IL-1β (A-C) and LDH (D-F) in the cell-free supernatants were measured. (G-I) BMDMs were infected with MAYV at a MOI of 5 (MOCK were used as control) for 24 hours infection and the cells were stained for active CASP1 (using FAM-YVAD) and analyzed as shown by the representative contour plots (G). The percentage (H) and integrated mean of fluorescence (iMFI) (I) of activated cells is shown. (J) After 24 hours of infection, supernatants (SN) and cellular extracts (CE) were harvested from MOCK or MAYV-infected BMDMs, and levels of cleaved CASP1 (p20) and IL-1β (p17) were detected by western blotting the SN and probing for the indicated proteins. As loading controls, levels of pro-caspase-1 and β-actin were also assessed in the CE. Data are shown as means ± standard deviation (SD) of triplicate samples (A-I) and are representative of three (A-J) independent experiments that yielded similar results. Statistical analysis was performed by student’s *t* test. *, *P* < 0.05.

Arboviruses such as CHIKV and ZIKV induce inflammasome activation [16,17,28–30]. We thus hypothesized that MAYV could also trigger inflammasome activation in macrophages. As an experimental *in vitro* model, BMDMs were employed, since these cells are a highly pure population of macrophages (S1A Fig). First, we primed BMDMs derived from C57BL/6 (WT) mice with PAM(3)CSK(4), a TLR2 agonist, for 4 hours and infected the macrophages with different multiplicities of infection (MOI) of CHIKV, ZIKV or MAYV. These three viruses induced IL-1β production (Fig 1A–1C) and LDH release (Fig 1D–1F) in a MOI-dependent manner. MAYV infection in unprimed BMDMs triggered *Il1b* expression at 3 and 6 hours after infection (S1B Fig), but it did not induce the release of significant levels of mature IL-1β (S1C Fig). Thus, priming with a TLR agonist is required to achieve robust release of IL-1β in macrophage cultures (S1C Fig). It is possible that *in vivo*, cytokines such as TNF-α are able to prime the cells. We assayed kinetics of IL-1β release by BMDMs and found a time-dependent secretion of IL-1β (S1D Fig). Additionally, we measured the kinetics of viral replication in unprimed and PAM(3)CSK(4) primed BMDMs. The virus has a fast replication cycle, as MAYV RNA levels reach its intracellular peak at 6 to 12 hours, while the extracellular peak is achieved at 12 hours after infection (S1E and S1F Fig). Priming BMDMs did not affect virus infectivity in BMDMs but affected virus output at 24 hours (S1E and S1F Fig).

Activation and processing of caspase-1 is a key event during inflammasome activation [23]. Therefore, we stained infected BMDMs with FAM-YVAD, a fluorescent dye that specifically binds to active Caspase-1 (CASP1). By FACS analysis, we gated in the FAM-YVAD+ population (S1G Fig) and found that MAYV induces robust CASP1 activation, as shown by the percentage of FAM-YVAD+ cells and the integrated mean of fluorescence (Fig 1G–1I). We determined that approximately 25% of the BMDMs are infected by MAYV (S2A and S2B Fig) or and 10% with CHIKV (S2D and S2E Fig). Interestingly, we found that approximately 50% of MAYV-infected BMDMs display active caspase-1 (S2C Fig), while 30% of CHIKV-infected BMDMs are FAM-YVAD+ (S2F Fig). Corroborating these data, we performed western blotting and detected the cleaved form of CASP1 (p20) and IL-1β (p17) in the supernatants of MAYV-infected BMDMs (Fig 1J). These data established that MAYV induces CASP1 activation and secretion of IL-1β in infected macrophages.

### The NLRP3 inflammasome is selectively triggered upon MAYV infection but is not important for viral replication in macrophages

**Fig 2:**
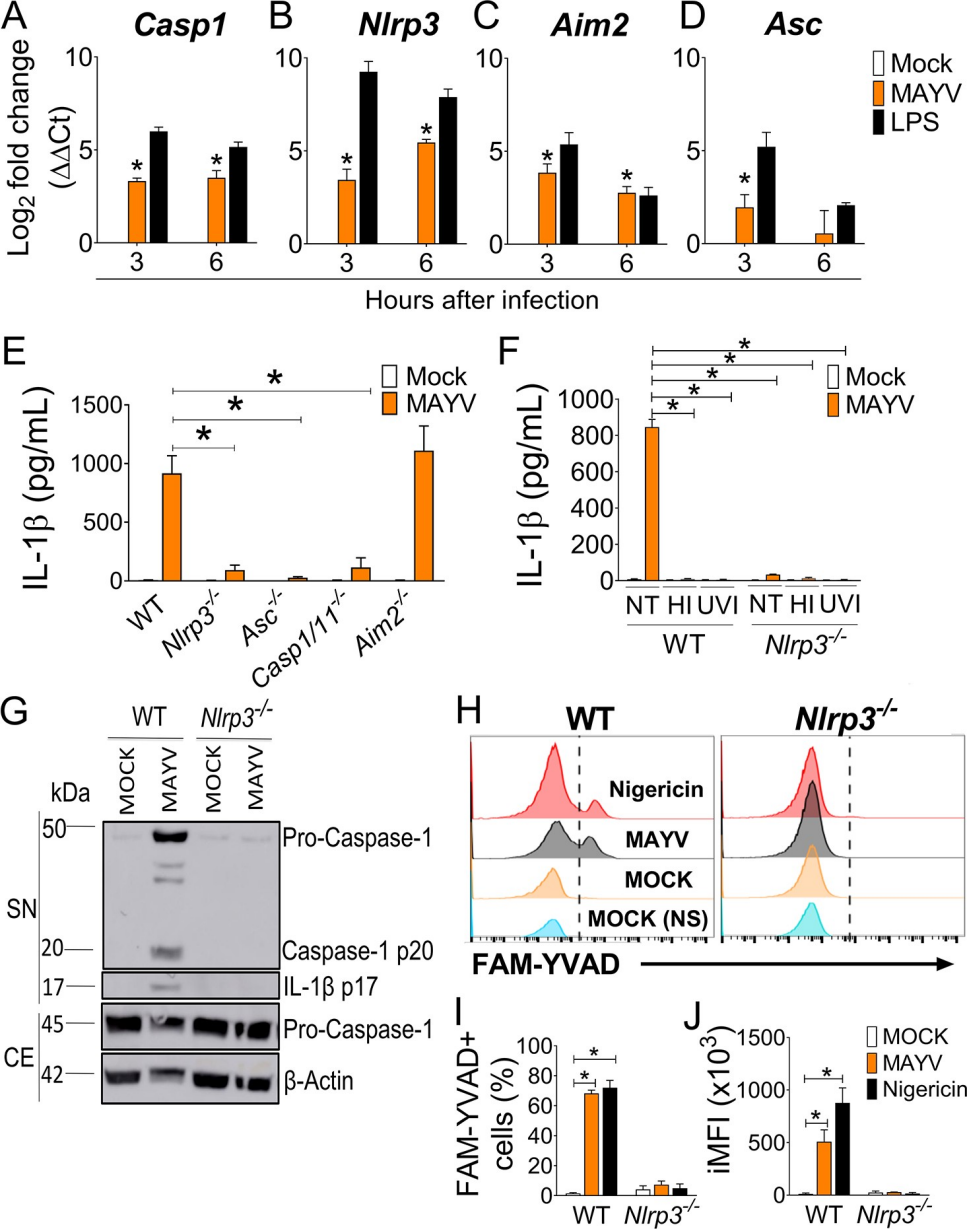
The NLRP3 inflammasome is activated in macrophages infected with MAYV. (A-D) WT bone marrow-derived macrophages (BMDMs) were infected with Mayaro virus (MAYV) at an MOI of 5 (MOCK was used as a control), or treated with RPMI medium (NI) or ultrapure LPS (500 ng mL^-1^) as negatives and positive controls, respectively. After 3 and 6 hours of infection, cells were lysed and the RNA was extracted for qPCR analysis of *Casp1* (A), *Nlrp3* (B), *Aim2* (C) *and Asc* (D). (E) PAM(3)CSK(4)-primed BMDMs derived from WT, *Nlrp3*^*–/–*^, *Asc*^*–/–*^, *Casp1/11*^*–/–*^and *Aim2*^*–/–*^mice were infected with MAYV at a MOI of 5. After 24 hours of infection, cell-free supernatants were harvested and IL-1β was quantified by ELISA. (F) Primed WT and *Nlrp3*^*–/–*^BMDMs were infected with either fresh MAYV, heat inactivated (HI) or UV-inactivated (UVI) virus. After 24 hours, the levels of IL-1β were measured by ELISA. (G) Western Blotting was performed in WT BMDMs after 24 hours of infection. Supernatants (SN) and cellular extracts (CE) were harvested from MAYV-infected (or MOCK) BMDMs, and levels of cleaved caspase-1 (p20) and IL-1β (p17) were detected in the SN. As loading controls, levels of pro-caspase-1 and β-actin were assessed in the CE. (H-J) WT and *Nlrp3*^*–/–*^BMDMs were infected with MAYV at a MOI of 5. After 24 hours of infection the cells were stained for active-caspase-1 with FAM-YVAD and analyzed as shown by the representative histograms (G). The percentage (H) and integrated mean of fluorescence (iMFI) (I) of activated cells is shown. Data shown are means ± SD of triplicate samples (A-E, G-I) and are representative of the data obtained from two (A-D, F) or three (E, G-H) independent experiments. Statistical analysis was performed by student’s *t* test. *, *P* < 0.05.

Different inflammasomes, including AIM2 and NLRP3, are activated in response to viral infections [28–35]. To address which inflammasome is activated upon MAYV infection, we assessed the mRNA expression of different inflammasome molecules in BMDMs by qPCR. At 3 and 6 hours post infection, MAYV induced increased expression of *Casp1* (Fig 2A), *Nlrp3* (Fig 2B), *Aim2* (Fig 2C) and *Asc* (Fig 2D). Next, we tested whether the AIM2 or NLRP3 inflammasomes were required for IL-1β release in macrophages. We observed robust IL-1β production in BMDMs from WT and *Aim2*^*–/–*^mice in response to infection with MAYV, but *Nlrp3*^*–/–*^, *Asc*^*–/–*^and *Casp1/11*^*–/–*^macrophages failed to induce IL-1β secretion (Fig 2E). Importantly, live virus is required for NLRP3 inflammasome activation, since neither heat-inactivated nor UV-irradiated MAYV were capable of inducing IL-1β release (Fig 2F). However, both NLRP3 and Casp1/11 were dispensable for LDH release (S3A Fig). By using western blot we found that NLRP3 was required for CASP1 (p20) and IL-1β (p17) cleavage in response to infection (Fig 2G). Accordingly, NLRP3 was also required for CASP1 activation measured by FAM-YVAD as shown by FACS analysis (Fig 2H–2J). To address whether NLRP3 activation by MAYV influenced viral replication, we infected *Nlrp3*^*–/–*^*and Casp1/11*^*–/–*^BMDMs with MAYV and found that NLRP3 inflammasome does not affect MAYV intracellular or extracellular RNA levels (S3B and S3C Fig).

### MAYV-induced NLRP3 activation requires ROS production and potassium efflux

**Fig 3:**
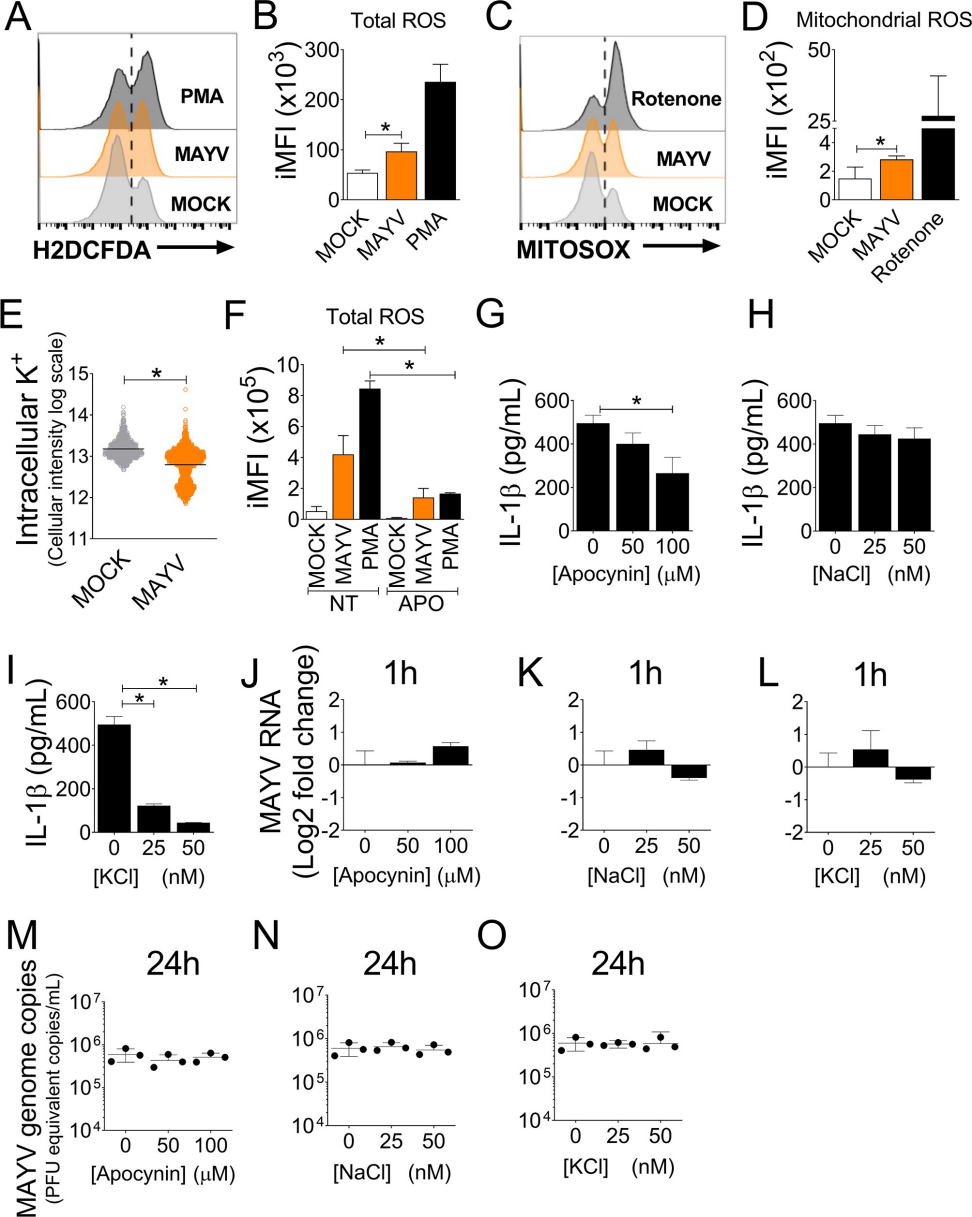
Potassium efflux, ROS and cathepsin B are required for efficient inflammasome activation. (A-D) WT bone marrow-derived macrophages (BMDMs) were infected with Mayaro virus (MAYV) at a MOI of 5 or MOCK infected for 90 minutes or treated with PMA or Rotenone (positive controls). Cells were stained for 30 minutes with, the fluorescent dyes H2DCFDA and MITOSOX, which stain total and mitochondrial ROS, respectively, harvested and analyzed by FACS. Representative histograms (A, C) and integrated mean of fluorescence (iMFI) data for each dye are shown (B, D). (E) After 2 hours of infection, BMDMs were incubated with APG-2 dye and the levels of intracellular potassium were determined as described in the methods. (F-I) Cells were left untreated or treated with apocynin (50 or 100 μM) for 1 hour and then infected with MAYV or treated with PMA (positive control). ROS production (F) was evaluated after treatment, and IL-1β levels (G) were assessed after 24 hours of infection by ELISA. BMDMs were primed with PAM(3)CSK(4) and incubated for 3 hours prior to infection with the indicated concentrations of NaCl (H) and KCl (I). After 24 hours, cell-free supernatants were collected and IL-1β levels were measured by ELISA. (J-O) MAYV RNA levels was determined by qPCR in Apocynin, KCl and NaCl-treated BMDMs after 1 (J-L) or 24 hours (M-O) of infection. Data shown are mean ± SD of quadruplicate samples and are representative of three independent experiments performed. Statistical analysis was performed by student’s *t* test. *, *P* < 0.05.

Cellular processes such as potassium efflux and ROS production are known to be important for NLRP3 inflammasome assembly and activation [12,23]. Therefore, we tested whether potassium efflux and ROS are induced by MAYV in BMDMs. MAYV triggered both total (Fig 3A and 3B) and mitochondrial ROS production (Fig 3C and 3D), as shown by the representative histograms and iMFI. In addition, MAYV infection also induced a significant decrease in intracellular potassium in BMDMs (Fig 3E). To address the importance of ROS production in NLRP3 inflammasome activation, we used apocynin, which inhibits NADPH oxidase activity [36]. We found that apocynin effectively blocked ROS production upon infection with MAYV or PMA stimulation (Fig 3F), and this effect resulted in a dose-dependent blockage of IL-1β secretion induced by MAYV infection (Fig 3G). We then tested the effect of K^+^ efflux on activation of the NLRP3 inflammasome in response to MAYV infection. We treated cells with KCl to increase extracellular K^+^ and used NaCl as a control [37]. Treatment with NaCl did not interfere in inflammasome activation in response to MAYV infection (Fig 3H). In contrast, KCl (Fig 3I) inhibited IL-1β release upon MAYV infection. Importantly, we measured viral infectivity and replication upon stimulation with the different treatments used and found that neither apocynin, nor NaCl or KCl affected viral infectivity at 1 hour (Fig 3J–3L), or viral load at 24 hours (Fig 3M–3O). Taken together, these results demonstrate that potassium efflux and ROS production are necessary for activation of the NLRP3 in response to MAYV infection.

### MAYV induces significant footpad swelling and pain in a mouse model of acute infection

**Fig 4:**
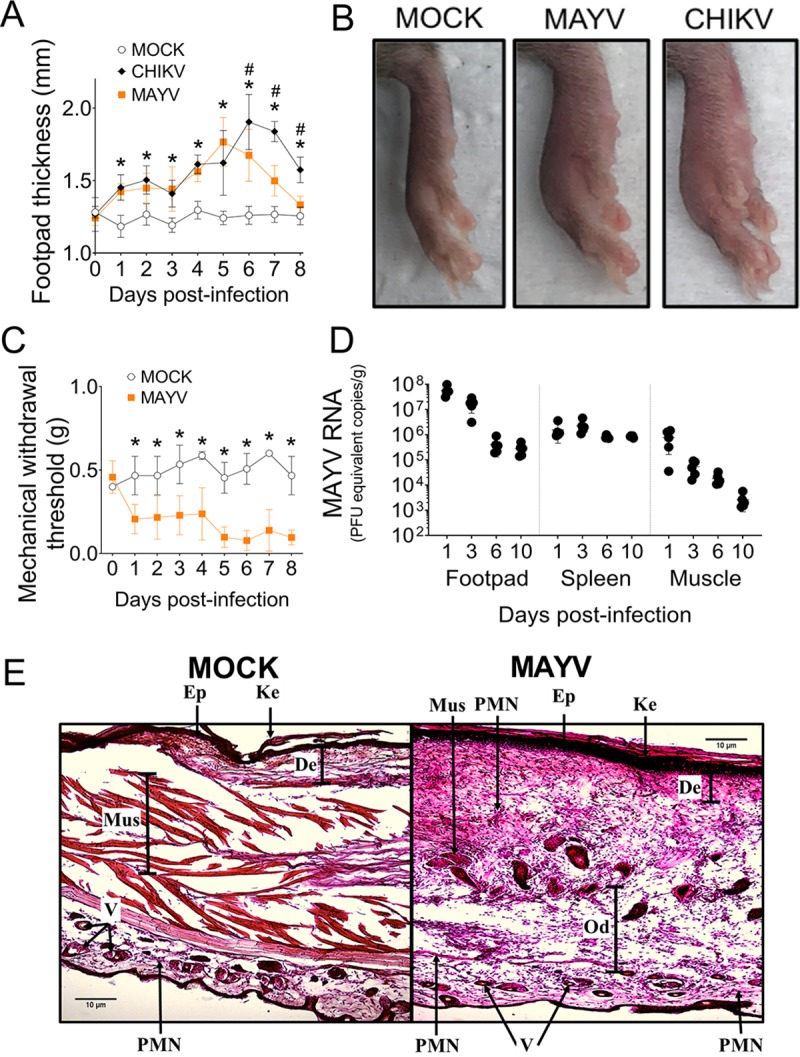
A mouse model to study acute inflammation by MAYV *in vivo*. WT mice (n = 5 per injected group) were injected subcutaneously into the footpad with 10 μL of conditioned media (mock), MAYV (10^6^ PFU) or CHIKV (10^7^ PFU). (A) Footpad thickness was measured daily (Footpad height) through the 8^th^ day of infection, and (B) representative images at day 6 after infection are shown. (C) The vonFrey test was used to measure pain in the paws of mock- and MAYV-injected mice. (D) Mice were euthanized at 1, 3, 6 and 10 days after infection and the amount of MAYV RNA was quantified in the footpad, muscle and spleen. (E) Histological examination (hematoxylin and eosin) of footpad sections from control (MOCK) or MAYV-infected mice after 6 days. Ke: keratin; Ep: epidermal layer; De: dermal layer; Mus: muscle; Od: oedema; V: Vessel; PMN: polymorphonuclear cell infiltration. Scale bars = 10 μM.

Mouse models of CHIKV infection are well established in the literature [38]. Of note, CHIKV injection into the footpad of mice induces acute inflammation, partially mimicking the pathogenesis of the disease in humans [17]. Patients infected by MAYV develop symptoms very similar to CHIKV-infected individuals [10,11,39]. Because of the similarity of clinical signs and previous reports showing that MAYV induce ankle or foot swelling in 4 weeks old C57BL/6 [27] or A129 mice [26], we tested whether MAYV was able to induce inflammation in the footpad of 6–8 weeks old WT C57BL/6 mice. We injected 10^6^ PFU of MAYV into the footpad of C57BL/6 mice, and footpad thicknesses were measured through eight days of infection. Conditioned media was used in mock infections as a negative control and 10^7^ PFU of CHIKV was used as a positive control. The magnitude of inflammation for both viruses was very similar, with the peak of MAYV-induced swelling at 5 to 6 days of infection (Fig 4A and 4B). We also performed the von-Frey test, which measures the mechanical withdrawal threshold, to assay pain in mice infected with MAYV. The results show that mice felt pain after 1 day of infection, until the end of the experiment at day 8 post infection (Fig 4C). The kinetics of MAYV replication were also measured in the infected footpad, spleen and leg muscle. Although viral copies were considerably high at 1 day after infection in all tissues analyzed, the viral load dropped considerably in the footpad and muscle up to day 10 of infection but remained stable in the spleen (Fig 4D). To evaluate the inflammatory infiltrate at the peak of infection, we infected WT mice with mock or MAYV and performed histological analyses of the footpads. Our results show that MAYV-induced footpad swelling was followed by a strong inflammatory infiltrate, composed primarily of myeloid cells (Fig 4E). Together, these results validate our *in vivo* model for MAYV-induced acute inflammation. This model resembles the well standardized CHIKV model of infection in the footpad, which is known to mimic infections in patients [17].

### NLRP3 inflammasome affects footpad swelling, inflammation and pain induction

**Fig 5:**
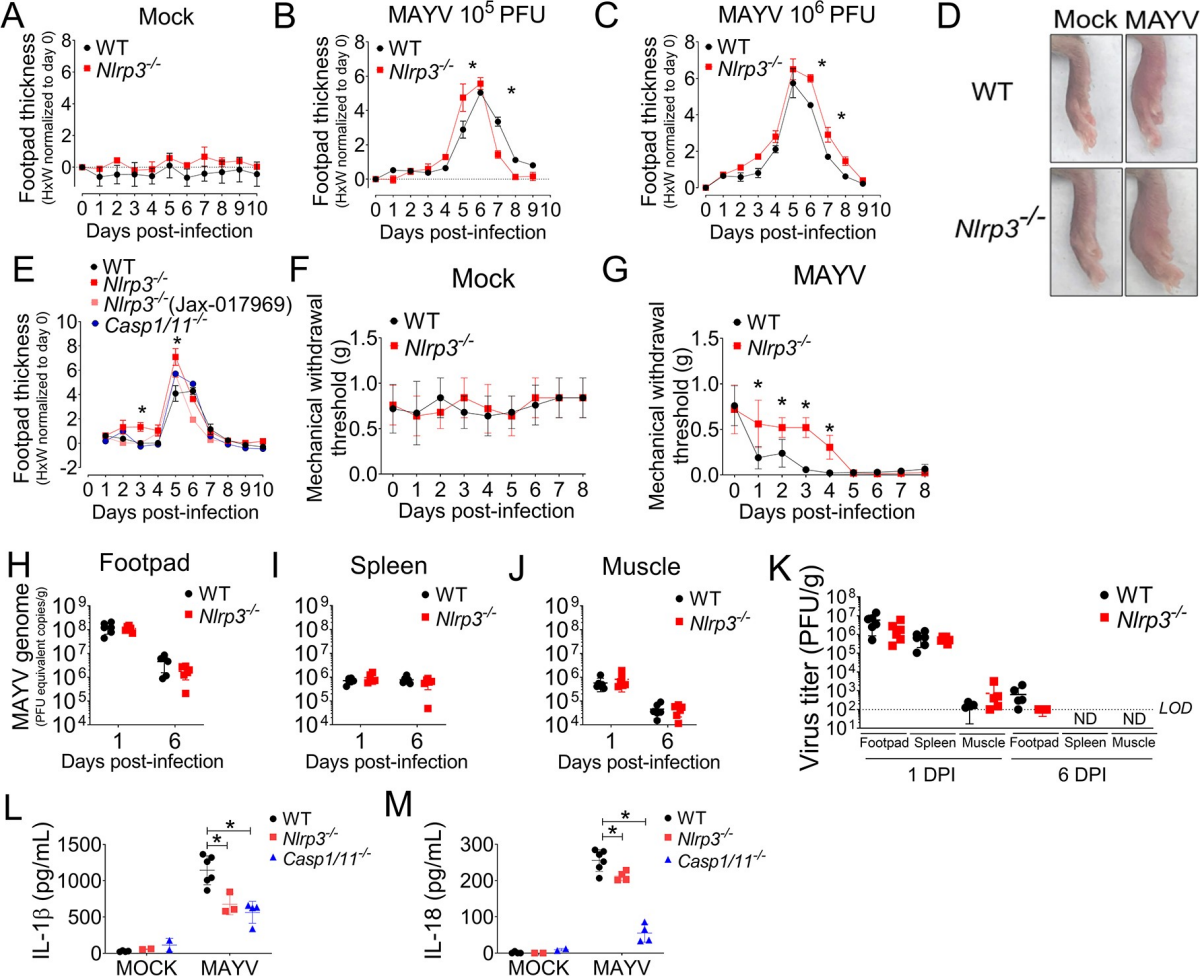
NLRP3 signaling participates in the pathogenesis of MAYV infection *in vivo*. WT and *Nlrp3*^*–/–*^mice (n = 5 per injected group) were injected subcutaneously into the footpad with 10 μL of MAYV (10^5^ or 10^6^ PFU) or mock infected. Footpad thickness height (H) X width (W) was measured daily through to the 10^th^ day of infection in mock (A) and MAYV-injected mice with 10^5^ PFU dose (B) or 10^6^ PFU dose (C). Representative images of paws at day 6 after infection are shown (D). WT, *Nlrp3*^*–/–*^(Genentech), *Nlrp3*^*–/–*^(Jackson), *Caspase1/11*^*-/-*^ mice (n = 5 per injected group) were injected subcutaneously into the footpad with 10 μL of MAYV (10^5^ PFU) or mock infected and footpad thickness was measured daily through to the 10^th^ day of infection (E). The von-Frey test was used to measure pain in the paws of mock (F) and MAYV-injected mice (G). (H-J) WT and *Nlrp3*^*–/–*^mice were euthanized at 1 and 6 days after infection and the amount of MAYV RNA was quantified in the footpad (H), muscle (I) and spleen (J). Virus titers were determined in the same organs by plaque assay (K) and are represented as mean ± SD. WT, *Nlrp3*^*–/–*^and *Casp1/11*^*-/-*^ mice were injected with Mock or MAYV for 5 days. Footpads were obtained, and both IL-1β (L) and IL-18 (M) were quantified in the homogenate’s supernatants. Data shown are representative of three (A-D) or two (E-J) independent experiments. Statistical analysis was performed by two-way ANOVA with Bonferroni’s multiple comparison test (M). Asterisks indicate significant differences (*P* < 0.05) between WT and *Nlrp3*^*–/–—*^infected groups, while in 5E asterisks indicate significant differences (*P* < 0.05) between WT and immune deficient-infected groups.

The NLRP3 inflammasome has been implicated in the development of CHIKV-induced inflammation in the footpad of injected mice [17]. We injected MAYV at 10^5^ or 10^6^ PFU dose in the footpad of WT and *Nlrp3*^*-/-*^ mice and observed that *Nlrp3*^*–/–*^mice had increased footpad swelling compared to WT mice at 5 days post infection with a dose of 10^5^ PFU and at 6 days post infection with a dose of 10^6^ PFU (Fig 5A, 5B and 5C). Representative images of infected WT and *Nlrp3*^*–/–*^mice are shown for both mock and MAYV-infected animals (Fig 5D). We next infected two independently generated *Nlrp3*^*–/–*^mice [40,41] and *Casp1/11*^*-/-*^ mice with 10^5^ PFU dose of MAYV and we observed that footpad swelling was significantly higher in these deficient strains compared to WT mice (Fig 5E). Of note, *Nlrp3*^*–/–*^mice showed reduced pain hypersensitivity at earlier time points upon infection but developed pain hypersensitivity similar to WT mice after 5 days of infection (Fig 5F and 5G).

Our data indicate that inflammasome signaling is involved with the pathogenesis of this acute model of MAYV infection. Thus, we tested whether the inflammasome plays a role in control of viral replication *in vivo*. To test this hypothesis, WT and *Nlrp3*^*–/–*^mice were injected with MAYV and euthanized at an early time point (1 day after infection) or at the peak of inflammation (6 days after infection), and virus RNA levels were measured by qPCR. NLRP3 deficiency did not alter viral loads in the footpad (Fig 5H), spleen (Fig 5I) or muscle (Fig 5J) of mice at 1 or 6 days post infection (Fig 5K). We next assessed whether activation of the NLRP3 inflammasome *in vivo* also occurs upon MAYV infection. We measured IL-1β and IL-18 levels in the supernatants obtained from footpad homogenates of Mock or MAYV-infected WT, *Nlrp3*^*-/-*^ and *Casp1/11*^*-/-*^ mice. These cytokines are produced upon infection in a NLRP3- and Caspase1/11-dependent manner (Fig 5L and 5M). Taken together, our results suggest that NLRP3 inflammasome activation by MAYV impacts the pathogenesis of an acute *in vivo* model of infection but does not play a role in viral control in macrophages or *in vivo*.

### NLRP3 inflammasome activation influence inflammatory infiltrate *in vivo*

**Fig 6:**
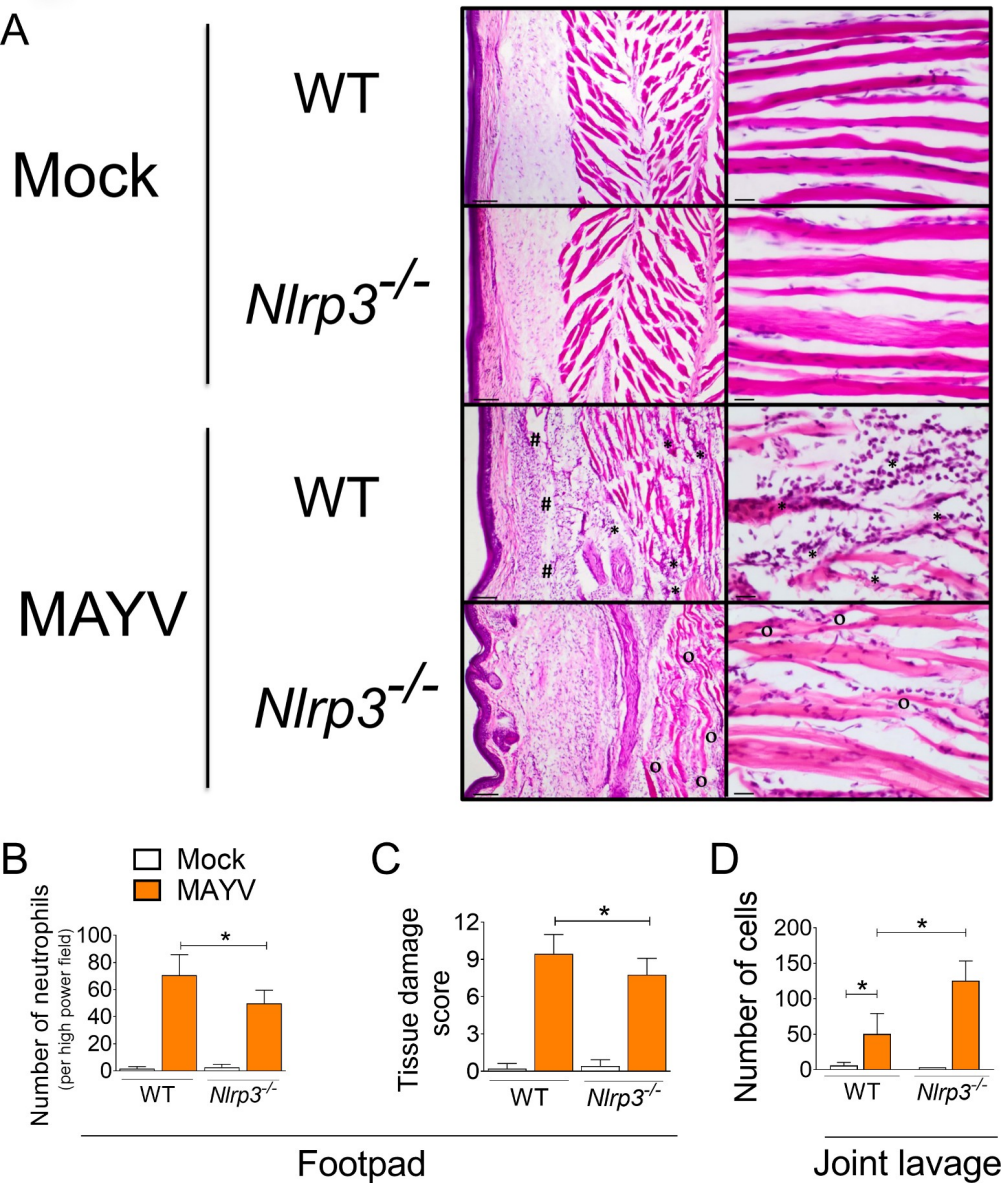
NLRP3 contributes to cell recruitment and tissue damage upon MAYV infection. WT and *Nlrp3*^*–/–*^mice (n = 3 per injected group) were injected subcutaneously into the footpad with 10 μL of MAYV (10^6^ PFU) or Mock. (A) Histological analysis of the mouse footpad histology sections after inoculation with Mock or MAYV. Inoculated feet were dissected, processed for histological analysis and stained with H&E. In MAYV-infected images, muscle necrosis and inflammation are shown by asterisks (*), whereas “#” labels regions of edema and inflammation of the subcutaneous layer and “o” points to muscle inflammation. Images are representative of at least 20 fields of view. Scale bars: 100 μM (Left panels); 20 μM (Right panels). (B, C) The number of neutrophils (B) and the tissue damage score (C) was quantified in the footpads of these mice. (D) Articular lavage was obtained from the knees and total cell numbers were quantified by Cytospin. Statistical analysis was performed by student’s t test (M). Data are representative of two independent experiments, and asterisks indicate significant differences (*P* < 0.05) between WT and *Nlrp3*^*–/–*^-infected groups.

To further investigate the differences in footpad swelling between WT and *Nlrp3*^*-/-*^ mice we performed histopathological analysis of mock and MAYV infected mice. Unexpectedly, the pronounced footpad swelling observed in *Nlrp3*^*-/-*^ mice was not followed by a greater infiltration of mononuclear cells and neutrophils in the tissue (Fig 6A and 6B). As expected, we found a robust neutrophil infiltration both in *Nlrp3*^*-/-*^ and WT mice infected with MAYV at 6 dpi (Fig 6A and 6B). In addition, MAYV-infected WT mice presented a higher tissue damage score when compared to *Nlrp3*^*-/-*^ mice (Fig 6C). In order to assess the inflammatory infiltrate in the joint tissue, we obtained articular lavages from the knees of mock and MAYV-injected mice. Although a considerable number of inflammatory cells was found in MAYV-infected WT mice, NLRP3 deficiency increased cellular infiltration into this joint tissue (Fig 6D).

**Fig 7:**
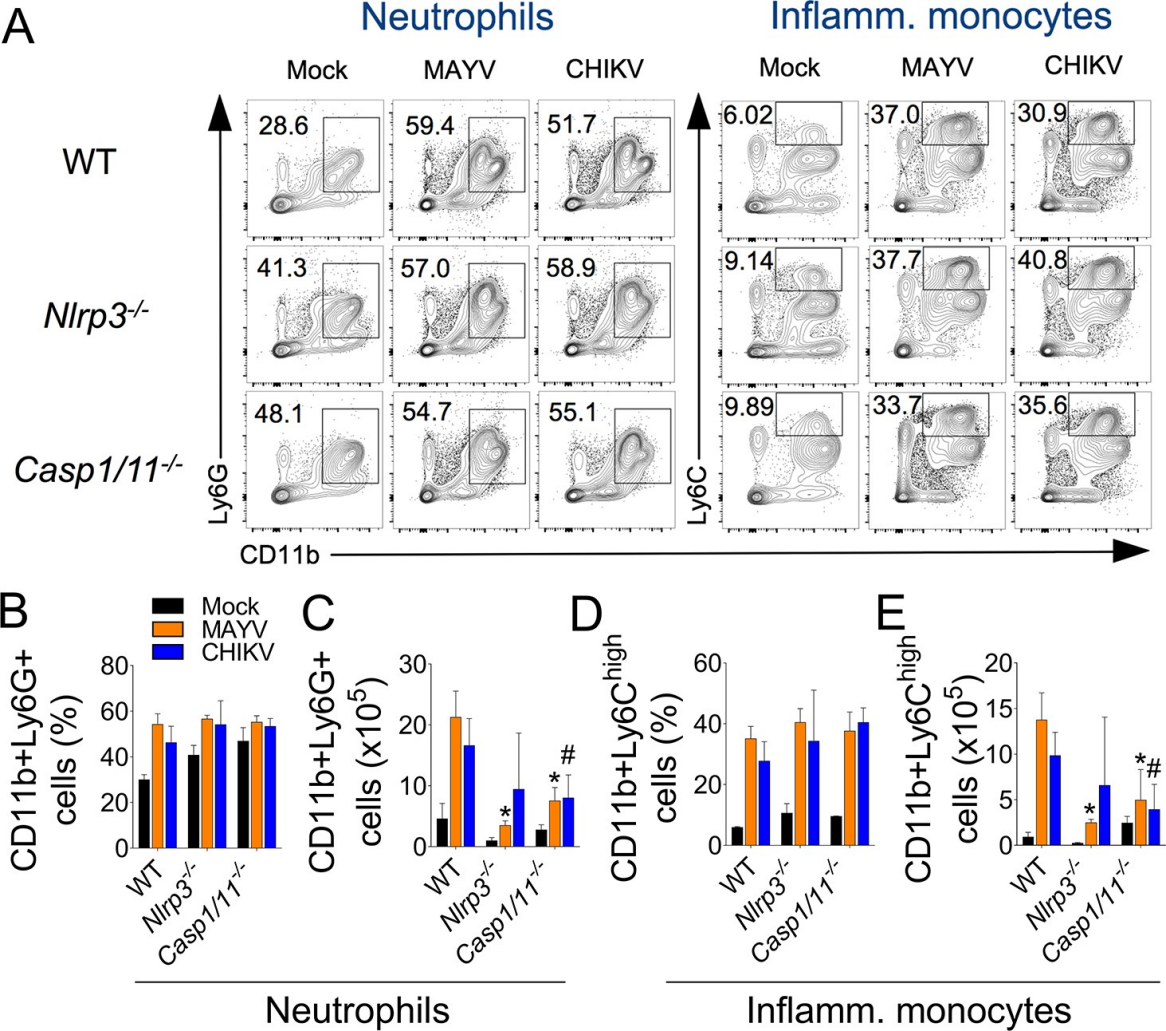
NLRP3 plays an important role in the recruitment of myeloid cells to the infected tissue. WT, *Nlrp3*^*–/–*^and *Casp1/11*^*–/–*^mice (n = 4 to 6 mice per injected group) were injected subcutaneously into the footpad with 10 μL of Mock, MAYV or CHIKV (10^6^ PFU). After 5 days of infection, footpads were removed and single cell suspensions were obtained for FACS analysis. (A) Representative contour plots showing the frequency of neutrophils (CD11b+Ly6G+) and inflammatory monocytes (CD11b+Ly6C^high^) in each experimental group. Frequencies of both types of cells (B,D) and their absolute numbers (C,E) are shown. Data are representative of two independent experiments. Statistical analysis was performed by student’s t test. Asterisks indicate significant differences (*P* < 0.05) between MAYV-infected *Nlrp3*^*-/-*^ or *Casp1/11*^*-/-*^ compared to WT mice, whereas # indicate differences (*P* < 0.05) between CHIKV-infected *Nlrp3*^*-/-*^ or *Casp1/11*^*-/-*^ compared to WT mice (C,E).

**Fig 8:**
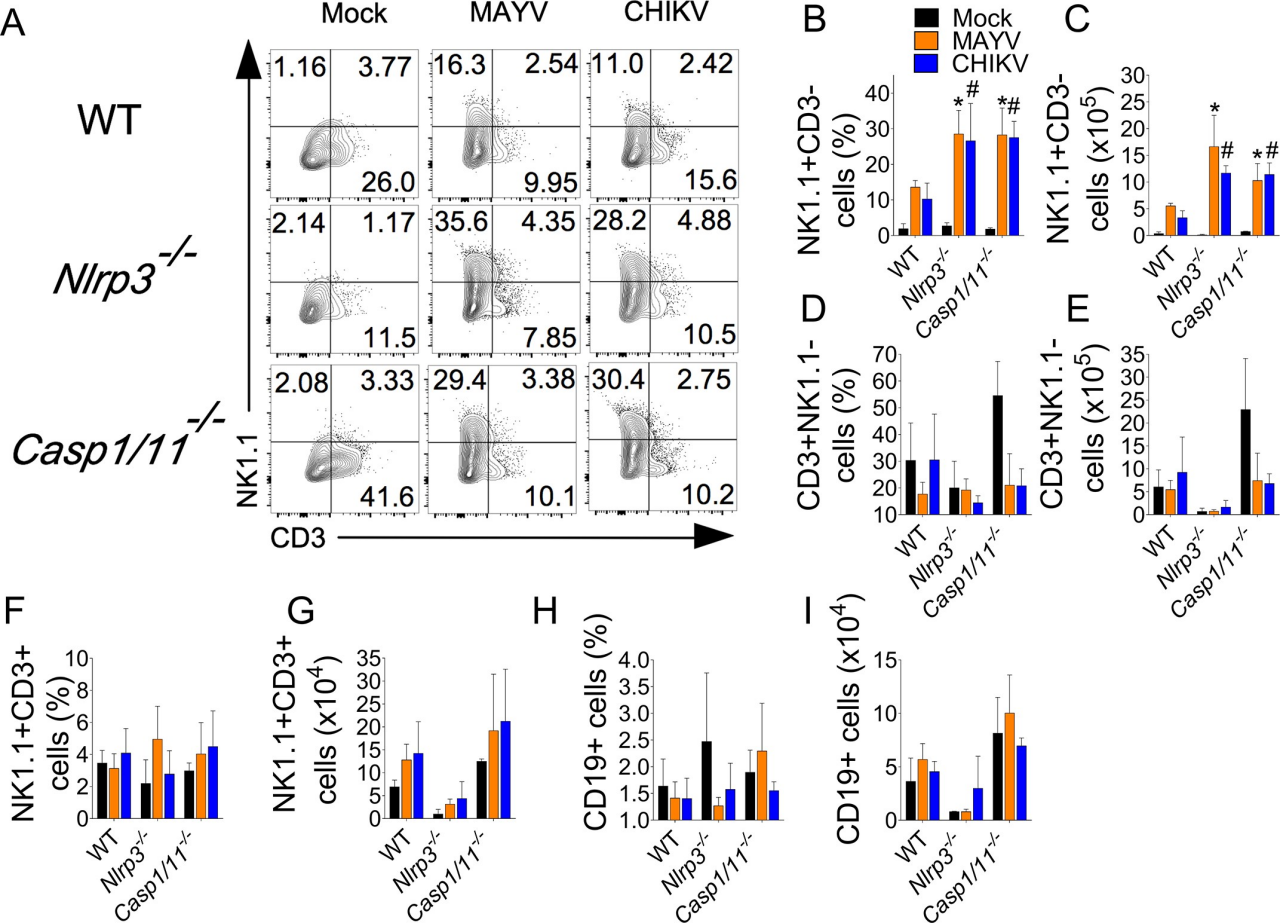
The inflammasome limits infiltration of NK cells into mice footpads. WT, *Nlrp3*^*–/–*^and *Casp1/11*^*–/–*^mice (n = 4 to 6 mice per injected group) were injected subcutaneously into the footpad with 10 μL of Mock, MAYV or CHIKV (10^6^ PFU). After 5 days of infection, footpads were removed and single cell suspensions were obtained for FACS analysis. (A) Representative contour plots showing the frequency of NK cells (CD45+NK1.1+CD3-) in each experimental group. Graphical quantification of the percentage of NK cells (B) and its absolute numbers (C) are shown. (D-I) T (CD45+CD3+NK1.1-), NKT cells (CD45+CD3+NK1.1+) and B cells (CD45+CD19+) were assessed in injected footpads. Frequencies of these types of cells (D,F,H) and their absolute numbers (E,G,I) are shown. Data are representative of two independent experiments. Statistical analysis was performed by student’s t test (M). Asterisks indicate significant differences (*P* < 0.05) between MAYV-infected *Nlrp3*^*-/-*^ or *Casp1/11*^*-/-*^ compared to WT mice, whereas # indicate differences (*P* < 0.05) between CHIKV-infected *Nlrp3*^*-/-*^ or *Casp1/11*^*-/-*^ compared to WT mice (B,D).

We next addressed the role of this inflammatory platform in the recruitment of specific cellular subsets found in the footpad of MAYV-infected mice (S4 Fig). Although the percentage of neutrophils (CD11b+Ly6G+) (Fig 7A and 7B) and inflammatory monocytes (CD11b+Ly6C^high^) (Fig 7A and 7D) infiltrating the tissue is not affected by the absence of NLRP3 or Casp1/11, all these populations decrease in absolute numbers compared to WT mice (Fig 7C and 7E, respectively). Besides myeloid cells, NK and T lymphocytes were also abundant in the footpad of MAYV-infected mice. Thus, we investigated whether NLRP3 play a role in the recruitment of these populations. We found that MAYV enhanced the percentage (Fig 8A and 8B) and absolute numbers (Fig 8C) of NK cells (CD45+NK1.1+CD3-) in WT mice and strikingly, the deficiency of NLRP3 and Caspase1/11 promoted an increased infiltration of this subset of cells to the infected tissue. We found that MAYV did not affect the percentage and absolute numbers of T (CD3+) (Fig 8D and 8E), NKT cells (CD45+NK1.1+CD3+) (Fig 8F and 8G) and B cells (CD19+) (Fig 8H and 8I). In addition, NLRP3 and Casp1/11 do not play a role in the recruitment of these populations. Taken together, our data demonstrate that NLRP3 activation by MAYV is important for recruitment of specific cells, induction of pain and inflammation in a mouse model of Mayaro infection.

### Proteins related to the NLRP3 inflammasome are increased in sera of MAYV-infected patients

**Fig 9:**
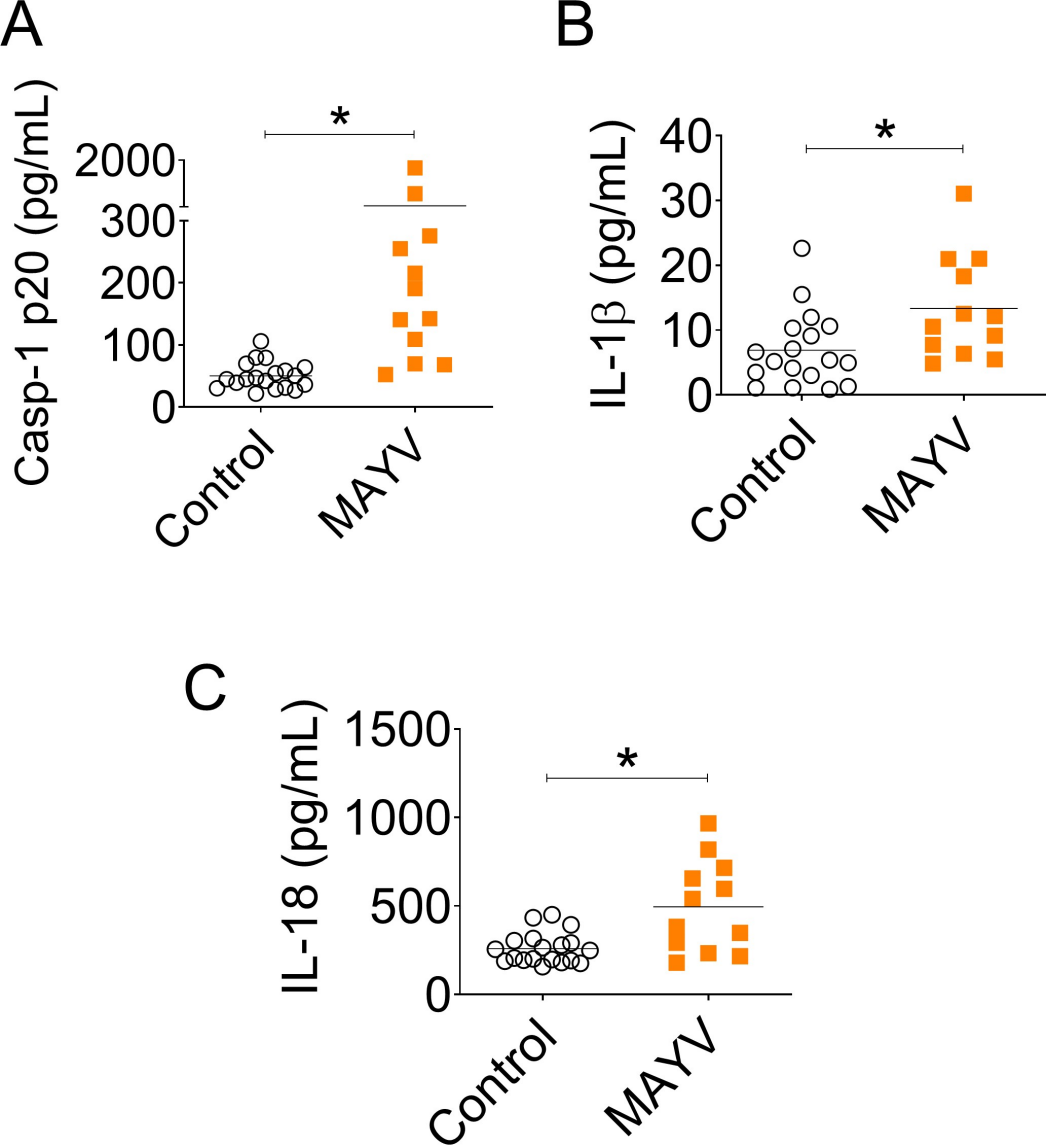
Caspase-1 p20, IL-1β and IL-18 are elevated in the serum of MAYV-infected patients in comparison to healthy individuals. Serum samples of 13 MAYV-infected patients and 19 healthy control samples were included. Samples were diluted 1:2 for caspase-1 p20 ELISA (A), 1:5 for IL-1β ELISA (B) and 1:3 for IL-18 ELISA (C). Data are represented as scatterplots showing means ± SEM of samples. Statistical analysis was performed by student’s t test. Asterisks indicate significant differences (*P* < 0.05) between MAYV-infected and control groups.

MAYV infection elicits robust immune responses in patients during acute and convalescent phase of the disease. In addition, secretion of pro-inflammatory immune mediators during the disease has been reported [11]. We evaluated inflammasome-related cytokines levels in sera of confirmed MAYV-infected patients presenting acute febrile illness for five days or less during the early phase of the disease. We found that active Caspase-1 (Caspase-1 p20) levels were higher in sera of infected individuals when compared to healthy controls samples (Fig 9A). Additionally, IL-1β and IL-18 levels in the sera of MAYV patients were higher than those found in healthy individuals (Fig 9B and 9C). These data indicate that MAYV infection is associated with the production of inflammasome-derived components such as Caspase-1 p20, IL-1β and IL-18, supporting our assertion that the NLRP3 inflammasome is important for MAYV clinical setting.

## Discussion

In this study, we evaluated whether MAYV activates the NLRP3 inflammasome and the possible mechanisms involved in the pathogenesis of this acute mouse model. Our findings demonstrate that MAYV triggers NLRP3 activation in macrophages, which have been implicated in the pathogenesis of many viruses [31,32,42–45]. Of note, MAYV induces IL-1β production similar to ZIKV and CHIKV, which are known to trigger the inflammasome [16,17,28–30]. Importantly, we showed that the NLRP3 inflammasome plays an important role during MAYV infection in our mouse model. In addition, the serum of MAYV-infected individuals contains elevated levels of active caspase-1, IL-1β and IL-18, supporting the participation of the NLRP3 inflammasome in the development of MAYV fever in humans.

Although the inflammasome is triggered by several viruses and plays an important role in the outcome of infections [31,32,42–45], our study is the first to identify the mechanisms of NLRP3 activation by an alphavirus. The finding that MAYV induces ROS production and potassium efflux, that are important to activate NLRP3, suggest that upon viral entry into the host cell, several PRRs may recognize the virus and affect cellular homeostasis, triggering mitochondrial and other organelle’s damage and inducing pore formation. IL-1β protein secretion was abrogated by inactivated MAYV, indicating that MAYV infection is required for the NLRP3 inflammasome activation. MAYV infection increased the levels of inflammasome-related mRNAs, including *Aim2*. However, infection did not activate the AIM2 inflammasome, since there was no alteration on secreted IL-1β levels using cells deficient in *Aim2*. Although MAYV infection induced an increase in steady-state *Il1b* transcript levels, priming with a TLR agonist prior to infection is needed for significant IL-1β secretion, in agreement with previously published literature with other viruses [45].

Besides cytokine production, cell death is another common consequence of inflammasome activation. However, our LDH release data suggest that cell death is NLRP3-independent. MAYV replication in BMDMs paralleled the kinetics of IL-1β production, which suggests that viral replication cycle itself may account for macrophage death. The cell death mechanism remains to be determined in future studies, since other forms of inflammatory cell death, such as necroptosis, might be triggered by MAYV.

Our *in vivo* model of MAYV acute infection demonstrates that NLR3 inflammasome signaling is associated with pain generation early during infection. This is consistent with previous studies in which NLRP3 inflammasome activation and pain induction in many disease models [46,47]. On the other hand, the finding that NLRP3 signaling was protective for the footpad swelling at the peak of MAYV inflammation was unexpected, since it has been demonstrated that inflammasome blockage is protective against CHIKV infection [48]. However, our FACS data demonstrate that NLRP3 is involved in the recruitment of neutrophils and inflammatory monocytes to the site of MAYV infection, while limiting infiltration of NK cells. Of note, it has been reported that caspase-1-specific antagonist Z-YVAD-FMK treatment leads to a reduction in cutaneous neutrophils recruitment during Semliki Forest virus infection [49]. The influx of inflammatory neutrophils are associated with worsened outcomes during CHIKV infection, since they support CCR2-dependent entry of myeloid cells and increase swelling during CHIKV infection [50]. However, while neutrophils initiate counterproductive responses at mosquito bites, they are required at later stages of disease to prevent mice from succumbing to Semliki Forest virus infection [49], demonstrating that they could have a dual role during alphavirus infections.

Interestingly, NK cells were shown to contribute to CHIKV pathogenesis [51], and may explain the increased footpad swelling in NLRP3-deficient mice infected with MAYV. During the acute phase of CHIKV infection there is an increased frequency of cytolytic NK cells in patients [52,53], which could indicate a role in the control of virus-infected target cells. Nevertheless, a greater infiltration of NK cells expressing granzyme B has been correlated with increased edema and footpad swelling during the early acute phase of CHIKV infection in a mouse model [54]. Depletion of NK cells significantly reduced the first peak of swelling during CHIKV infection (3dpi), while not affecting the second peak of swelling (6dpi) of the biphasic pattern of footpad swelling [54]. Although the greater infiltration of NK cells could lead to the increased swelling in NLRP3-deficient mice, we cannot not discard the participation of the inflammasome in edema formation, cell activation and cytokine production, given that we found no roles for NLRP3 in controlling viral titers, a finding consistent with previous studies [48,55]. Inflammasome may present a dual role in alphavirus infections, having protective or deleterious effects. Against other viruses, such as influenza, the NLRP3 activation can be either protective or detrimental depending on the stage of infection and virus load [31,32,55,56]. In addition, mosquito saliva was shown to enhance inflammasome activation and Semliki Forest virus disease [49].

The NLRP3 inflammasome may act alongside other pathways during the development of disease in MAYV-infected patients. We found higher levels of caspase-1, IL-1β and IL-18 in MAYV-infected patients during the acute phase of the disease, showing that the NLRP3-inflammasome might be relevant during MAYV infection in the clinical setting. Recent epidemics of arboviral diseases, such as ZIKV and CHIKV, have emphasized the need for health agencies to anticipate potential emerging pathogens transmitted by arthropods. MAYV infections have been emerging in South America representing a potential threat to public health. There is an urgent need to elucidate the basic processes of MAYV pathogenesis, which is essential to better understand the disease and manage patients. In this scenario, this study advances toward the understanding of the pathogenesis of the disease caused by this virus. We identified the NLRP3 inflammasome as an important pathway related to MAYV pathogenesis, paving the way to future studies exploring the mechanisms governing this disease.

## Materials and methods

### Ethics statement

The care of the mice was in compliance with the institutional guidelines on ethics in animal experiments; approved by CETEA (Comissão de Ética em Experimentação Animal da Faculdade de Medicina de Ribeirão Preto, approved protocol number 014/2016). CETEA follow the Brazilian national guidelines recommended by CONCEA (Conselho Nacional de Controle em Experimentação Animal). All proceedings involving human samples and data were approved by the Julio Muller University Hospital ethics committee (process number 1.164.656). This institution follows the recommendation from CONEP (Comissão Nacional de Ética em Pesquisa). Samples were obtained with written informed consent from each patient. All participants were adults.

### Viruses

Virus strains used in this study comprised MAYV BeAr 20290, CHIKV BzH1 and ZIKV ZikaSPH2015. Genomic sequences of these 3 viruses were deposited in NCBI (National Center for Biotechnology Information) database under the GenBank accession numbers KT754168, KT581023 and KU321639 respectively [57,58]. Virus stocks were produced after infecting Vero cells (ATCC CCL-81) with a MOI of 0.1 PFU and cultured in DMEM with 2% heat-inactivated FCS, 1% glutamine and 1% Pen-Strep. The supernatant was collected after 2–3 days of infection for MAYV and CHIKV and 5 days for ZIKV, clarified by centrifugation to remove cell debris (5500g) and aliquots were kept at −80°C. Conditioned media used for mock infections was prepared from uninfected Vero cells in a similar manner. Virus stocks were titrated by plaque assay in Vero cells using 10-fold serial dilutions of virus stocks [59]. To inactivate MAYV by ultraviolet light (UV), the virus was dispersed in a tissue culture dish, and a compact UV lamp was placed directly above the dish for 30 minutes. Heat-inactivated MAYV was prepared by incubating the virus at 70°C for 15 minutes. Conditioned media used for mock infections was treated in a similar manner. Virus complete inactivation was verified by the lack of virus plaques after titration by standard plaque assays on Vero cells.

### MAYV infected patient samples

The patients samples used were previously described and confirmed for MAYV acute infection by RT-nested-PCR, sequencing, virus isolation and IgM and IgG detection [60,61]. The samples were obtained from May 2015 to March 2016 from patients of Mato Grosso state of Brazil, presenting acute febrile illness with symptoms for five days or less. Patients age presented a median of 40 years old (ranging from 24 to 66 years) and most of them were women (77%). Control serum samples were collected from healthy subjects not infected with arboviruses.

### Mice

Mice used were C57BL/6J mice (JAX 000664), *Nlrp3*^*−/−*^ [41], *Casp1/11*^*−/−*^ [62], *Aim2*^*−/−*^ [63], *Nlrc4*^*−/−*^ [64], *Asc*^*−/−*^ [65] and *Il1r*^*−/−*^ (JAX 003245), all on a C57BL/6 mouse background. When indicated, the JAX *Nlrp3*^*−/−*^ (JAX stock #017969) was also used [40]. All mice were bred and maintained under specific-pathogen-free conditions at the Animal Facilities of the Medical School Ribeirão Preto (FMRP-USP). For *in vitro* experiments bone marrow were collected from 6–12 week old female and/or male mice. The *in vivo* experiments were conducted using 6–8 week old mice.

### *In vivo* infections and footpad swelling

6–8 week old male mice (n = 5 mice per group) were inoculated subcutaneously (10 μL) in the ventral side of the footpad with 10^5^ or 10^6^ PFU of MAYV. Mock-infected mice were inoculated with the conditioned media (10 μL). MAYV-induced footpad swelling was assessed every 24 h by measuring height (H) or the height (H) X width (W) of the perimetatarsal area of the hind foot using Kincrome digital vernier calipers. The total area (H x W) was normalized for day 0 measurements.

### Bone marrow–derived macrophage preparation and *in vitro* infections

BMDMs were prepared using tibia and femur from 6- to 12-week-old mice as previously described [66]. Briefly, progenitor cells were isolated by flushing femurs and tibia with cold sterile incomplete RPMI 1640 (Gibco). The cells were then cultured in differentiation medium: RPMI 1640 supplemented with 20% heat-inactivated FCS and 30% L-929 cell-conditioned medium (LCCM) as a source of M-CSF. After 7 days in culture, BMDMs were harvested and seeded at the required density for each experiment. An MOI of 5 and 24 hours of infection were used in the experiments *in vitro* using BMDMs unless otherwise stated in the figure legends.

### Cytokine measurements by ELISA

For *in vitro* cytokine determination, BMDMs were seeded overnight at a density of 2 × 10^5^ cells/well in 48-well plates and prestimulated with 300 ng/ml of PAM(3)CSK(4) (Invivogen) for 4 h, and subsequently infected with MAYV. The cytokines in the supernatants were assayed using a mouse IL-1β ELISA kit (BD Biosciences) according to the manufacturer’s instructions. For *in vivo* cytokine determination, footpads were processed and the supernatants from total homogenates were obtained. The levels of IL-1β and IL-18 in the homogenates were detected using a mouse IL-1β ELISA kit (BD Biosciences) and a mouse IL-18 ELISA kit (RD-MBL), respectively, according to the manufacturer’s instructions. Detection of IL-1β, IL-18 and Caspase 1 (p20 subunit) in human serum samples was accomplished by using human IL-1β ELISA kit II (BD Biosciences), human total IL-18 (RD) and human caspase-1 ELISA kit (RD) respectively.

### Analysis of inflammasome related genes expression by qPCR

Total RNA was extracted from 1x10^6^ BMDMs using RNeasy Mini Kit (Qiagen), according to the manufacturer’s instructions. RNA concentrations were determined in a NanoDrop One spectrophotometer (Thermo Fisher Scientific) and 1 μg of the extracted RNA was used for cDNA conversion using the iScriptTM cDNA Synthesis kit (BIO-RAD) in a thermal cycler. Primers used were *Asc*Forward: 5’-CCAGTGTCCCTGCTCAGAGT-3’; *Asc*Reverse: 5’-TCATCTTGTCTTGGCTGGTG-3’*; Casp1*Forward: 5’-AGATGCCCACTGCTGATAGG-3’; *Casp1*Reverse: 5’-TTGGCACGATTCTCAGCATA-3’; *Nlrp3*Forward: 5’-GTGGTGACCCTCTGTGAGGT-3’; *Nlrp3*Reverse: 5’-TCTTCCTGGAGCGCTTCTAA-3’*; Aim2*Forward: 5’-TCTGTCCTCAAGCTAAGCCTCA-3’; *Aim2*Reverse: 5’-GTGACAACAAGTGGATCTTTCTGTA-3’; *Il1b*Forward: 5’-CCAAGCAACGACAAAATACC-3’; *Il1b*Reverse: 5’-GTTGAAGACAAACCGTTTTTCC-3’; *Hprt*Forward: 5’-CAGTCCCAGCGTCGTGATTA-3’; *Hprt*Reverse: 5’-GGCCTCCCATCTCCTTCATG-3’. The quantification of *Asc*, *Casp1*, *Nlrp3 and Il1b* produtcs was performed using 50 ng of cDNA, 10 μM of each primer and PowerUp SYBR Green Master Mix (Applied Biosystems) according to the manufacturer’s instructions. The reactions were performed in the QuantStudio 3 Real-Time PCR System (Applied Biosystems). Quantitation was performed by normalizing target gene mRNA levels to Hprt (hypoxanthine guanine phosphoribosyl transferase) levels, and infected sample values are expressed relative to the mean of mock values. Statistical significance between-groups was calculated with ΔCT values that provides the estimates of ΔΔCT values (Log2 fold change)[67,68].

### Western blot analysis

A total of 10^7^ BMDMs were seeded in 6-well plates overnight and then primed with 300 ng/ml PAM(3)CSK(4) (InvivoGen, tlrl-pms) for 4 hours prior to infection with MAYV or mock infection. After 24 hours the supernatants were harvested and proteins were precipitated with ice-cold 50% trichloroacetic acid followed by acetone. Cells were lysed in RIPA buffer (10 mM Tris-HCl, pH 7.4, 1 mM EDTA, 150 mM NaCl, 1% Nonidet P-40, 1% deoxycholate and 0.1% SDS) in the presence of a protease inhibitor cocktail (complete, Roche). Precleared lysates and supernatants were boiled in Laemmli sample buffer, resolved by SDS-PAGE and transferred (Semidry Transfer Cell, Bio-Rad) to a 0.22-μm nitrocellulose membrane (GE Healthcare). The membranes were blocked in Tris-buffered saline (TBS) with 0.01% Tween-20 and 5% nonfat dry milk. Rat monoclonal antibody to CASP1 p20 (1:250, Genentech, 4B4), goat antibody to IL-1β p-17 (1:200, Sigma Aldrich, I3767), mouse anti-β-Actin (1:1000, C4, Santa Cruz sc-47778) and specific horseradish peroxidase–conjugated antibodies (1:3,000, KPL, 14-16-06 and 14-13-06) were diluted in blocking buffer for the incubations. Enhanced chemiluminescence luminol reagent (GE Healthcare) was used for antibody detection.

### Endogenous caspase-1 staining using FAM-YVAD–FMK and intracellular virus staining

Active CASP1 was measured by fluorochrome inhibitor of caspases assay (YVAD-FLICA, ImmunoChemistry Technologies), a green fluorescent dye that binds specifically to active CASP1. Briefly, 10^6^ BMDMs were seeded in 12-well plates overnight and then infected with MAYV (MOI of 5) or mock infected for 24 hours. As a positive control, we used 20 μM of Nigericin (Sigma-Aldrich) for 40–60 minutes. After that, cells were harvested and stained for 1 h with YVAD-FLICA, following the manufacturer's instructions. For intracellular virus staining, BMDMs were infected with either MAYV or CHIKV (MOI of 5) or mock-treated for 8 hours. After that, cells were fixed in paraformaldehyde 4% and permeabilized with 0,1% saponin. Cells were incubated for 1 hour with mouse hyperimmune sera to MAYV, CHIKV or isotype control and with FAM-YVAD fluorescent dye, following the manufacturer's instructions. Mouse hyperimmune sera to MAYV strain BeAr20290 and to CHIKV strain S27-African had their reactivity previously confirmed by indirect immunofluorescence assay [69]. Secondary antibody anti-mouse stained with Alexa-594 was added and incubated for 40 minutes. Cells were then detached from the plates and the data were acquired on a FACS ACCURI C6 flow cytometer (BD Biosciences) and analyzed with FlowJo software (Tree Star).

### Measurement of potassium efflux

Intracellular concentration of potassium was determined by fluorescence emission of asante potassium green-2 (APG-2, TEFLabs). BMDMs (2×10^4^) were seeded in black, clear-bottom 96-well plates, and treated with PAM(3)CSK(4) for 4 hours, then infected with MAYV (MOI of 5). After 2 hours of infection, BMDMs were incubated with 5 μM APG-2 in RPMI without FBS and phenol red for 30 min. The cells were washed with PBS, and RPMI without phenol red was replaced. Nine images per well were recorded at 40× magnification with the ImageXpress Micro High-Content Imaging System and processed with MetaXpress High-Content Image Acquisition and Analysis (Molecular Devices).

### Measurement of ROS production

To detect intracellular and mitochondrial ROS production, we seeded 10^6^ BMDMs in 12-well plates overnight. Cells were infected with MAYV (MOI of 5) or stimulated with PMA (200 ng/ml) or rotenone (50 μM) for 90 minutes. Next, H2DCFDA (10 μM) and MitoSOX Red dye (2.5 μM) were added to the cells for 30 min at 37°C, and then they were harvested and analyzed by flow cytometry. The data were acquired on a FACS ACCURI C6 flow cytometer (BD Biosciences) and analyzed with the FlowJo software (Tree Star).

### Inhibition of potassium efflux and ROS production

BMDMs were primed with 300 ng/ml of PAM(3)CSK(4) (InvivoGen) for 4 hours, treated for 2 hours with 0–50 nM of NaCl or KCl, and then infected with MAYV (MOI of 5). To inhibit NADPH oxidase, cells were treated with 50 or 100 μM apocynin for 1 hour prior to infection. After 24 hours of infection, supernatants were collected and the levels of IL-1β were measured by ELISA (BD Biosciences). The effect of these treatments on virus infectivity and virus production were measured at 1 hour, intracellularly, and 24 hours, extracellularly, after infection with MAYV (MOI of 5) by viral RNA detection by qPCR as described below.

### Cell death assay

BMDMs were seeded overnight in 48-well plates (2x10^5^ cells/well), primed with 300 ng/mL of PAM(3)CSK(4) for 4 hours, and then infected for 24 hours with MAYV, CHIKV or ZIKV in RPMI1640 medium without phenol red, 15 mM HEPES and 2 g/l NaHCO_3_ supplemented with 2% FBS. At the end of the infection, supernatants were collected and assayed using the CytoTox 96 Non-Radioactive Cytotoxicity Assay (Promega) following the manufacturer’s instructions. Cells were incubated with 9% Triton X-100 (Fisher Scientific) for 15 min as a positive control for complete cell lysis. The percentage of LDH release was calculated as (mean OD value of sample / mean OD value of Triton X-100 control sample) × 100, and is shown in the figures as the percentage of cell death compared to Triton X-100.

### Quantitation of viral loads in tissues by qPCR and plaque assay

At the times indicated after infection, mice were euthanized and perfused by intracardial injection with PBS. Tissues were dissected, weighed, and homogenized in sterile PBS using a TissueLyser II (Qiagen). The homogenized tissues were diluted 1:3 in serum-free DMEM (Thermo Fisher Scientific) and seeded onto a monolayer of Vero cells in 24-well plates. The plaque assay was performed as described previously for the quantitation of viral stocks [59]. For viral RNA quantification, aliquots from the same samples used for plaque assay were extracted using QIAamp Viral RNA Mini Kit (QIAGEN) according to the manufacturer's recommendations. RT-qPCR was performed in one-step using TaqMan Fast Virus 1-Step Master Mix (Applied Biosystems), following the manufacturer's recommendations. Primers and probe used were designed to detect a 99 bp region of MAYV Nsp2 gene (*Nsp2*Forward: 5’-GGCATTGCATCCTTTAGCGG-3’; *Nsp2*Reverse: 5’-GGGAGTAGAACACGGCCATC-3’; Probe: FAM TACCCACAAAGGTCGTGCAGGGCGATACCAAG BHQ1). The reaction was performed in the QuantStudio 3 Real-Time PCR System (Applied Biosystems). Standard curves were generated using titrated virus stocks. qPCR results were normalized to the amount of virus in PFU. Each sample was assayed in duplicate.

### Histological analysis

After 6 days of infection, animals were deeply anesthetized with ketamine and xylazine and perfused through the ascending aorta with PBS, followed by 4% paraformaldehyde (PFA). After perfusion the mouse footpad tissue was immediately removed (skin and muscle). Pieces of the footpad tissue were post-fixed for 24 h in PFA and then replaced with 20% sucrose for 4 days. The tissues were embedded in Tissue-Tek O.C.T. compound and sectioned at 15μm thickness. The specimens were dehydrated in ascending grades of ethyl alcohol, cleared in xylene and stained with Harris haematoxylin and eosin (H&E) for histopathology [70]. Image acquisition was performed by using light microscopy (DM6000B; Leica Microsystems, Buffalo Grove, IL, USA). From each slide, twenty representative photographs were randomly taken (magnification 400x) for analysis of the histological changes. In each high-power field, the degree of tissue damage was determined by a modified score [71]: (1) tissue edema (2) infiltration or aggregation of inflammatory cells, and (3) muscle necrosis. Each item was graded according to the following five-point scale: 0, no damage; 1, minimal damage; 2, mild damage; 3, moderate damage; 4, severe damage. The degree of tissue disease was assessed by the sum of scores ranging from 0 to 12 for each high-power field. In addition, quantification of subcutaneous tissue thickness was performed by histomorphometric analysis using ImageJ software (National Institutes of Health, U.S.A.), and the total number of polymorphonuclear neutrophils infiltrated into the tissues was counted.

### Synovial lavage

Knee cavities were surgically opened, and synovial lavage was obtained by flushing the joint cavity with 10μl. Cytospin preparations were acquired by Cytospin 4 (Thermo Scientific) with 70 μl of diluted joint lavage per slide and centrifugation at 200 r.p.m. for 7 min. The slides were air-dried and Giemsa-stained (Laborclin, Pinhais, Brazil) and counted under a light microscope with a 40X objective to determine the total numbers of cells in the lavage fluid.

### Immunophenotyping of footpads

Mice were infected as detailed in *In vivo infections and footpad swelling section*, and after 5 days of infection for MAYV or 6 days for CHIKV, the animals were sacrificed, and footpads were removed. Footpads homogenates were obtained after 2 hours incubation in collagenase VIII at 1mg/mL and passed thought a 70 μm cell strainer. Cells were counted and plated in 96-well U bottom. Cells were blocked with Fc Block (BD Biosciences) and then stained for flow cytometry analysis. The antibodies employed were anti-CD3ε-PerCP (BioLegend), anti-CD19-APC (BioLegend), anti-NK1.1-FITC (BioLegend), anti-CD45-APC (BD Biosciences), anti-CD11b-FITC (BioLegend), anti-Ly6G-PerCP (BioLegend) and anti-Ly6C-PE (BioLegend). Cells were also stained with a viability dye (Live/Dead fluorescent dye, Pacific Blue, Life Technologies). Samples were acquired on a FACS Verse flow cytometer (BD Biosciences) and analyzed with FlowJo software (Tree Star).

### Behavioral nociceptive tests

To evaluate the mechanical nociceptive threshold, mice were placed on an elevated wire grid and the plantar surface of the ipsilateral hind paw was stimulated perpendicularly with a series of von Frey filaments (Stoelting, Chicago, IL, USA) with logarithmically increasing stiffness (0.008–2.0 g) and the basal mechanical withdrawal threshold was measured one day before infection. Each one of these filaments was applied for approximately 3–4 s to induce a paw-withdrawal reflex. The weakest filament able to elicit a response was taken to be the mechanical withdrawal threshold. The log stiffness of the filament is reported as log10 of the mass of the filament in mg and ranged from 0.903 (8 mg or 0.008 g) to 3.0 (1000 mg or 1 g) [72,73].

### Statistical analysis

Data were plotted and analyzed with GraphPad Prism 8.1 software (GraphPad, San Diego, California). Log-transformed values for viral load data were used for statistical comparisons. For comparisons of multiple groups, two-way analysis of variance (ANOVA) followed by the Bonferroni's post test were used. The differences in values obtained for two different groups were determined using an unpaired, two-tailed Student’s t test with a 95% confidence interval. Differences were considered statistically significant when the *P* < 0.05.

## Supporting information

S1 Fig MAYV activates the inflammasome in BMDMs. (A) FACS analysis was employed to demonstrate the percentage of mature BMDMs in our culture at 6 days of differentiation. Initially, cells were gated to exclude doublets (FSC-A, FSC-W), and then to exclude debris (SSC-A, FSC-A). The double-positive population represents mature macrophages (CD11b+F4/80+). (B) WT BMDMs were infected with MAYV at a MOI of 5, or treated with RPMI medium (NI), or condoned media (mock), or ultrapure LPS (500 ng/ml) as negative and positive controls, respectively. After 3 or 6 hours of infection, cells were lysed and RNA was extracted for qPCR analysis of *Il1b*. Macrophages were primed with PAM(3)CSK(4) (C,D) or left non-primed (NP) (C) for 4 hours and infected with MAYV at a MOI of 5. After 1–24 (D) or 24 hours (C), levels of IL-1β in cell-free supernatants were quantified by ELISA. (E,F) Non-treated (NT) and PAM(3)CSK(4)-primed BMDMs were infected with MAYV at a MOI of 0.2, and at the indicated times total RNA was extracted from the cellular extracts and supernatants to evaluate MAYV intercellular or extracellular mRNA levels by qPCR. (G) Gating strategy used in FACS analyses of FLICA assays employing FAM-YVAD. First, cells were gated to exclude doublets (FSC-A/FSC-H), then to exclude other debris (SSC-A/FSC-A). Data are represented as the means ± SD of triplicate samples (A, B) and are representative of at least two independent experiments that yielded similar results. Statistical analysis was performed by student’s *t* test. Asterisks indicate statistically significant differences between MOCK and MAYV groups. *P < 0.05.
(TIF)

S2 Fig Fraction of infected BMDMs displaying active caspase-1 upon MAYV or CHIKV. (A) FACS analysis was employed to demonstrate the percentage of BMDMs infected by either MAYV (A-C) or CHIKV (D-F) after 8 hours of infection. The representative contour plots showing the percentages in each plot are shown (A,D), alongside the quantification of MAYV (B) or CHIKV-infected cells (E). MAYV (C) or CHIKV-infected cells (F) were also gated for active caspase-1 (FAM-YVAD). IC: isotype control. Data are represented as the means ± SD of triplicate samples and are representative of two independent experiments that yielded similar results. Statistical analysis was performed by student’s *t* test. Asterisks indicate statistically significant differences between MOCK and infected groups. *P < 0.05.
(TIF)

S3 Fig MAYV-induced cell death and viral replication in macrophages are NLRP3-independent events. (A) WT, *Nlrp3*^*–/–*^and *Casp1/11*^*–/–*^BMDMs were primed with PAM(3)CSK(4) and then infected with MAYV at a MOI of 5 or mock infected for 24 hours. Cells were also treated with Nigericin (positive control) for 40–60 minutes. Cell-free supernatants were then harvested and LDH release was quantified as previously described. (B) WT, *Nlrp3*^*–/–*^and *Casp1/11*^*–/–*^BMDMs were infected with MAYV at a MOI of 0.2, and at the indicated times total RNA was extracted from the cellular extracts and supernatants to evaluate MAYV intracellular (B) or extracellular (C) mRNA levels by qPCR. Data are represented as the means ± SD of quadruplicate samples and are representative of two independent experiments. Statistical analysis was performed by student’s *t* test. Asterisks indicate statistically significant differences between groups. *P < 0.05.
(TIF)

S4 Fig Gating strategy used for immunophenotyping analysis of footpad. Representative contour plot showing the frequencies of different subsets of hematopoietic cells (CD45+) infiltrating the footpads of MAYV-infected mice. First, cells were gated to exclude doublets (FSC-A/FSC-H), then to exclude other debris (SSC-A/FSC-A). Live cells were selected and hematopoietic cells (CD45+) were gated. Then myeloid (CD11b+), NK (NK1.1+CD3-) and NKT cells (NK1.1+CD3+) and T and B cells (CD3+CD19+) were gated.
(TIF)

## References

[1] Hotez PJ, Murray KO. Dengue, West Nile virus, chikungunya, Zika-and now Mayaro? PLoS Negl Trop Dis. 2017;11: e0005462 10.1371/journal.pntd.0005462 28859086 PMC5578481

[2] Seymour C, Peralta PH, Montgomery GG. Serologic evidence of natural togavirus infections in Panamanian sloths and other vertebrates. Am J Trop Med Hyg. 1983;32: 854–861. 10.4269/ajtmh.1983.32.854 6309027

[3] Auguste AJ, Liria J, Forrester NL, Giambalvo D, Moncada M, Long KC, et al Evolutionary and ecological characterization of mayaro virus strains isolated during an outbreak, Venezuela, 2010. Emerg Infect Dis. 2015;21: 1742–1750. 10.3201/eid2110.141660 26401714 PMC4593426

[4] Medlin S, Deardorff ER, Hanley CS, Vergneau-Grosset C, Siudak-Campfield A, Dallwig R, et al Serosurvey of selected arboviral pathogens in free-ranging, two-toed sloths (Choloepus hoffmanni) and three-toed sloths (Bradypus variegatus) in Costa Rica, 2005–07. J Wildl Dis. 2016;52: 883–892. 10.7589/2015-02-040 27479900 PMC5189659

[5] Terzian ACB, Auguste AJ, Vedovello D, Ferreira MU, Da Silva-Nunes M, Sperança MA, et al Isolation and characterization of Mayaro virus from a human in Acre, Brazil. Am J Trop Med Hyg. 2015;92: 401–404. 10.4269/ajtmh.14-0417 25510721 PMC4347347

[6] Lednicky J, De Rochars VMB, Elbadry M, Loeb J, Telisma T, Chavannes S, et al Mayaro Virus in Child with Acute Febrile Illness, Haiti, 2015. Emerg Infect Dis. 2016;22: 2000–2002. 10.3201/eid2211.161015 27767924 PMC5088037

[7] Mavian C, Rife BD, Dollar JJ, Cella E, Ciccozzi M, Prosperi MCF, et al Emergence of recombinant Mayaro virus strains from the Amazon basin. Sci Rep. Springer US; 2017;7: 1–11. 10.1038/s41598-017-07152-5 28821712 PMC5562835

[8] Chen R, Mukhopadhyay S, Merits A, Bolling B, Nasar F, Coffey LL, et al ICTV Virus Taxonomy Profile: Togaviridae. J Gen Virol. 2018;99: 761–762. 10.1099/jgv.0.001072 29745869 PMC12662122

[9] Long KC, Ziegler SA, Thangamani S, Hausser NL, Kochel TJ, Higgs S, et al Experimental transmission of Mayaro virus by Aedes aegypti. Am J Trop Med Hyg. 2011;85: 750–7. 10.4269/ajtmh.2011.11-0359 21976583 PMC3183788

[10] Taylor SF, Patel PR, Herold TJS. Recurrent arthralgias in a patient with previous Mayaro fever infection. South Med J. 2005;98: 484–5. 10.1097/01.SMJ.0000145879.14102.F4 15898531

[11] Santiago FW, Halsey ES, Siles C, Vilcarromero S, Guevara C, Silvas JA, et al Long-Term Arthralgia after Mayaro Virus Infection Correlates with Sustained Pro-inflammatory Cytokine Response. PLoS Negl Trop Dis. 2015;9: e0004104 10.1371/journal.pntd.0004104 26496497 PMC4619727

[12] Guo H, Callaway JB, Ting JP-Y. Inflammasomes: mechanism of action, role in disease, and therapeutics. Nat Med. 2015;21: 677–687. 10.1038/nm.3893 26121197 PMC4519035

[13] de Carvalho RVH, Soares SMA, Gualberto ACM, Evangelista GCM, Duque JAM, Ferreira AP, et al Plasmodium berghei ANKA infection results in exacerbated immune responses from C57BL/6 mice displaying hypothalamic obesity. Cytokine. 2015;76: 545–548 10.1016/j.cyto.2015.01.025 26239414

[14] Hagar JA, Powell DA, Aachoui Y, Ernst RK, Miao EA. Cytoplasmic LPS activates caspase-11: Implications in TLR4-independent endotoxic shock. Science. 2013; 341: 1250–253 10.1126/science.1240988 24031018 PMC3931427

[15] de Carvalho RVH, Silva ALN, Santos LL, Andrade WA, de Sá KSG, Zamboni DS. Macrophage priming is dispensable for NLRP3 inflammasome activation and restriction of Leishmania amazonensis replication. J Leuko Biol. 2019;106:631–640. 10.1002/JLB.MA1118-471R 31063608

[16] Wang W, Li G, De W, Luo Z, Pan P, Tian M, et al Zika virus infection induces host inflammatory responses by facilitating NLRP3 inflammasome assembly and interleukin-1β secretion. Nat Commun. 2018;9:106 10.1038/s41467-017-02645-3 29317641 PMC5760693

[17] Chen W, Foo SS, Zaid A, Teng TS, Herrero LJ, Wolf S, et al Specific inhibition of NLRP3 in chikungunya disease reveals a role for inflammasomes in alphavirus-induced inflammation. Nat Microbiol. 2017;2: 1435–1445. 10.1038/s41564-017-0015-4 28848230

[18] Srikiatkhachorn A, Mathew A, Rothman AL. Immune-mediated cytokine storm and its role in severe dengue. Semin Immunopathol. Seminars in Immunopathology; 2017;39: 563–574. 10.1007/s00281-017-0625-1 28401256 PMC5496927

[19] O’Neill LAJ, Golenbock D, Bowie AG. The history of Toll-like receptors—redefining innate immunity. Nat Rev Immunol. Nature Publishing Group; 2013;13: 453–60. 10.1038/nri3446 23681101

[20] Kagan, Jonathnan C.; Barton GM. Emerging Principles Governing Signal Transduction by Pattern- Recognition Receptors. Cold Spring Harb Perspect Biol. 2016;33: 395–401. 10.1038/nbt.3121 25395297 PMC4355268

[21] de Carvalho RVH, Andrade WA, Lima-Junior DS, Dilucca M, de Oliveira C V., Wang K, et al Leishmania Lipophosphoglycan Triggers Caspase-11 and the Non-canonical Activation of the NLRP3 Inflammasome. Cell Rep. ElsevierCompany.; 2019;26: 429–437.e5. 10.1016/j.celrep.2018.12.047 30625325 PMC8022207

[22] Lupfer C, Malik A, Kanneganti TD. Inflammasome control of viral infection. Curr Opin Virol. 2015;12: 38–46 10.1016/j.coviro.2015.02.007 25771504 PMC4470791

[23] Broz P, Dixit VM. Inflammasomes: mechanism of assembly, regulation and signalling. Nat Rev Immunol. 2016;16: 407–420. 10.1038/nri.2016.58 27291964

[24] Martinon F, Burns K, Tschopp J. The Inflammasome: A molecular platform triggering activation of inflammatory caspases and processing of proIL-β. Mol Cell. 2002;10: 417–426. 10.1016/S1097-2765(02)00599-3 12191486

[25] Rathinam VAK, Fitzgerald KA. Inflammasome Complexes: Emerging Mechanisms and Effector Functions. Cell. 2016;165: 792–800. 10.1016/j.cell.2016.03.046 27153493 PMC5503689

[26] Weise WJ, Hermance ME, Forrester N, Adams AP, Langsjoen R, Gorchakov R, et al A Novel Live-Attenuated Vaccine Candidate for Mayaro Fever. 2014;8 10.1371/journal.pntd.0002969 25101995 PMC4125120

[27] Fox JM, Long F, Edeling MA, Fremont DH, Rossmann MG, Diamond MS, et al Broadly Neutralizing Alphavirus Antibodies Bind an Epitope on E2 and Inhibit Entry and Egress Article Broadly Neutralizing Alphavirus Antibodies Bind an Epitope on E2 and Inhibit Entry and Egress. Cell. Elsevier; 2015;163: 1095–1107. 10.1016/j.cell.2015.10.050 26553503 PMC4659373

[28] Ekchariyawat P, Hamel R, Bernard E, Wichit S, Surasombatpattana P, Talignani L, et al Inflammasome signaling pathways exert antiviral effect against Chikungunya virus in human dermal fibroblasts. Infect Genet Evol. Elsevier B.V.; 2015;32: 401–408. 10.1016/j.meegid.2015.03.025 25847693

[29] He Z, Chen J, Zhu X, An S, Dong X, Yu J, et al NLRP3 Inflammasome Activation Mediates Zika Virus-Associated Inflammation. J Infect Dis. 2018;217: 1942–1951. 10.1093/infdis/jiy129 29518228

[30] Khaiboullina SF, Uppal T, Sarkar R, Gorzalski A, St Jeor S, Verma SC. ZIKV infection regulates inflammasomes pathway for replication in monocytes. Sci Rep. Springer US; 2017;7: 1–14. 10.1038/s41598-017-16072-3 29167459 PMC5700238

[31] Allen IC, Scull MA, Moore CB, Holl EK, Mcelvania-tekippe E, Taxman DJ, et al Article The NLRP3 Inflammasome Mediates In Vivo Innate Immunity to Influenza A Virus through Recognition of Viral RNA. Immunity. Elsevier Ltd; 2009;30: 556–565. 10.1016/j.immuni.2009.02.005 19362020 PMC2803103

[32] Thomas PG, Dash P, Aldridge JR, Ellebedy AH, Reynolds C, Funk AJ, et al Article The Intracellular Sensor NLRP3 Mediates Key Innate and Healing Responses to Influenza A Virus via the Regulation of Caspase-1. Immunity. Elsevier Ltd; 2009;30: 566–575. 10.1016/j.immuni.2009.02.006 19362023 PMC2765464

[33] Rathinam VAK, Jiang Z, Waggoner SN, Sharma S, Cole LE, Waggoner L, et al The AIM2 inflammasome is essential for host defense against cytosolic bacteria and DNA viruses. Nat Immunol. Nature Publishing Group; 2010;11: 395–402. 10.1038/ni.1864 20351692 PMC2887480

[34] Hornung V, Ablasser A, Charrel-Dennis M, Bauernfeind F, Horvath G, Caffrey DR, et al AIM2 recognizes cytosolic dsDNA and forms a caspase-1-activating inflammasome with ASC. Nature. Nature Publishing Group; 2009;458: 514–518. 10.1038/nature07725 19158675 PMC2726264

[35] Yogarajah T, Ong KC, Perera D, Wong KT. AIM2 Inflammasome-Mediated Pyroptosis in Enterovirus A71-Infected Neuronal Cells Restricts Viral Replication. Sci Rep. Springer US; 2017;7: 1–16. 10.1038/s41598-017-05589-2 28724943 PMC5517550

[36] Lima-Junior DS, Mineo TWP, Calich VLG, Zamboni DS. Dectin-1 Activation During L. amazonensis Phagocytosis Prompts Syk-Dependent Reactive Oxygen Species Production To Trigger Inflammasome Assembly and Restriction of Parasite Replication. J Immunol. 2017; 199:2055–2068 10.4049/jimmunol.1700258 28784846

[37] Lima-Junior DS, Costa DL, Carregaro V, Cunha LD, Silva ALN, Mineo TWP, et al Inflammasome-derived IL-1β production induces nitric oxide-mediated resistance to Leishmania. Nat Med. 2013;19: 909–15. 10.1038/nm.3221 23749230

[38] Haese NN, Broeckel RM, Hawman DW, Heise MT, Morrison TE, Streblow DN. Animal models of chikungunya virus infection and disease. J Infect Dis. 2016;214: S482–S487. 10.1093/infdis/jiw284 27920178 PMC5137241

[39] Pinheiro FP, Freitas RB, Travassos da Rosa JF, Gabbay YB, Mello WA, LeDuc JW. An outbreak of Mayaro virus disease in Belterra, Brazil. I. Clinical and virological findings. Am J Trop Med Hyg. 1981;30: 674–81. 10.4269/ajtmh.1981.30.674 6266263

[40] Brydges SD, Mueller JL, McGeough MD, Pena CA, Misaghi A, Gandhi C, et al Inflammasome-Mediated Disease Animal Models Reveal Roles for Innate but Not Adaptive Immunity. Immunity. Elsevier Ltd; 2009;30: 875–887. 10.1016/j.immuni.2009.05.005 19501000 PMC2759865

[41] Mariathasan S, Weiss DS, Newton K, McBride J, O’Rourke K, Roose-Girma M, et al Cryopyrin activates the inflammasome in response to toxins and ATP. Nature. 2006;440: 228–232. 10.1038/nature04515 16407890

[42] McAuley JL, Tate MD, MacKenzie-Kludas CJ, Pinar A, Zeng W, Stutz A, et al Activation of the NLRP3 Inflammasome by IAV Virulence Protein PB1-F2 Contributes to Severe Pathophysiology and Disease. PLoS Pathog. 2013;9 10.1371/journal.ppat.1003392 23737748 PMC3667782

[43] Wang W, Xiao F, Wan P, Pan P, Zhang Y, Liu F, et al EV71 3D Protein Binds with NLRP3 and Enhances the Assembly of Inflammasome Complex. PLoS Pathog. 2017;13: 1–30. 10.1371/journal.ppat.1006123 28060938 PMC5245909

[44] Rajan J V., Rodriguez D, Miao EA, Aderem A. The NLRP3 Inflammasome Detects Encephalomyocarditis Virus and Vesicular Stomatitis Virus Infection. J Virol. 2011;85: 4167–4172. 10.1128/JVI.01687-10 21289120 PMC3126243

[45] Ito M, Yanagi Y, Ichinohe T. Encephalomyocarditis Virus Viroporin 2B Activates NLRP3 Inflammasome. PLoS Pathog. 2012;8:e1002857 10.1371/journal.ppat.1002857 22916014 PMC3415442

[46] Jia M, Wu C, Gao F, Xiang H, Sun N, Peng P, et al Activation of NLRP3 inflammasome in peripheral nerve contributes to paclitaxel-induced neuropathic pain. Mol Pain. 2017;13: 1–11. 10.1177/1744806917719804 28714351 PMC5562344

[47] Goldberg EL, Asher JL, Molony RD, Shaw AC, Zeiss CJ, Wang C, et al β-Hydroxybutyrate Deactivates Neutrophil NLRP3 Inflammasome to Relieve Gout Flares. Cell Rep. ElsevierCompany.; 2017;18: 2077–2087. 10.1016/j.celrep.2017.02.004 28249154 PMC5527297

[48] Chen W, Foo S-S, Zaid A, Teng T-S, Herrero LJ, Wolf S, et al Specific inhibition of NLRP3 in chikungunya disease reveals a role for inflammasomes in alphavirus-induced inflammation. Nat Microbiol. Springer US; 2017;10:1435–1445. 10.1038/s41564-017-0015-4 28848230

[49] Pingen M, Bryden SR, Pondeville E, Schnettler E, Kohl A, Merits A, et al Host Inflammatory Response to Mosquito Bites Enhances the Severity of Arbovirus Infection. Immunity. 2016;44: 1455–1469. 10.1016/j.immuni.2016.06.002 27332734 PMC4920956

[50] Major LD, Larcher T, Gardner J, Poo YS, Schroder WA, Le TT, et al CCR2 Deficiency Promotes Exacerbated Chronic Erosive Neutrophil-Dominated Chikungunya Virus Arthritis. J Virol. 2014;88: 6862–6872. 10.1128/JVI.03364-13 24696480 PMC4054367

[51] Maucourant C, Petitdemange C, Yssel H, Vieillard V. Control of Acute Arboviral Infection by Natural Killer Cells. Viruses. 2019;11: 131 10.3390/v11020131 30709036 PMC6410043

[52] Thanapati S, Das R, Tripathy AS. Phenotypic and functional analyses of NK and NKT-like populations during the early stages of chikungunya infection. Front Microbiol. 2015;6: 1–11. 10.3389/fmicb.2015.00895 26388848 PMC4555083

[53] Leroy EM, Debré P, Petitdemange C, Béziat V, Becquart P, Vieillard V, et al Unconventional Repertoire Profile Is Imprinted during Acute Chikungunya Infection for Natural Killer Cells Polarization toward Cytotoxicity. PLoS Pathog. 2011;7: e1002268 10.1371/journal.ppat.1002268 21966274 PMC3178577

[54] Lee WWL, Her Z, Rénia L, Tan JJL, Teo T-H, Gallian P, et al Caribbean and La Réunion Chikungunya Virus Isolates Differ in Their Capacity To Induce Proinflammatory Th1 and NK Cell Responses and Acute Joint Pathology. J Virol. 2015;89: 7955–7969. 10.1128/JVI.00909-15 25995257 PMC4505608

[55] Tate MD, Ong JDH, Dowling JK, McAuley JL, Robertson AB, Latz E, et al Reassessing the role of the NLRP3 inflammasome during pathogenic influenza A virus infection via temporal inhibition. Sci Rep. 2016;6: 1–8. 10.1038/srep27912 27283237 PMC4901306

[56] Ichinohe T, Lee HK, Ogura Y, Flavell R, Iwasaki A. Inflammasome recognition of influenza virus is essential for adaptive immune responses. J Exp Med. 2009;206: 79–87. 10.1084/jem.20081667 19139171 PMC2626661

[57] Espósito DLA, da Fonseca BAL. Complete Genome Sequence of Mayaro Virus (Togaviridae, Alphavirus) Strain BeAr 20290 from Brazil. Genome Announc. 2015;3: 141660 10.1128/genomeA.01372-15 26679574 PMC4683219

[58] Cunha MS, Alves L, Rocco M, Maeda Y, Silva G, Nogueira JS, et al First complete genome sequence of the Zika virus released. Genome Announc. 2016; 4: e00032–16. 10.1128/genomeA.00032-16 26941134 PMC4777745

[59] Juarez D, Long KC, Aguilar P, Kochel TJ, Halsey ES. Assessment of plaque assay methods for alphaviruses. J Virol Methods. Elsevier B.V.; 2013;187: 185–189. 10.1016/j.jviromet.2012.09.026 23085307

[60] de Souza Costa MC, Siqueira Maia LM, de Souza VC, Gonzaga AM, de Azevedo VC, Martins LR, et al Arbovirus Investigation in Patients From Mato Grosso During Zika and Chikungunya Virus Introdution in Brazil, 2015–2016. Acta Trop. Elsevier B.V.; 2018; 2015–2016. 10.1016/j.actatropica.2018.12.019 30552880

[61] Fumagalli MJ, Marciel de Souza W, Romeiro MF, Costa MC de S, Slhessarenko RD, Figueiredo LTM. Development of an Enzyme-Linked Immunosorbent assay to detect antibodies targeting the recombinant envelope protein 2 of Mayaro virus. J Clin Microbiol. 2019;57: e01892–18. 10.1128/JCM.01892-18 30787146 PMC6498026

[62] Kuida K, Lippke JA, Ku G, Harding MW, Livingston DJ, Su MSS, et al Altered cytokine export and apoptosis in mice deficient in interleukin-1β converting enzyme. Science (80-). 1995;267: 2000–2003. 10.1126/science.7535475 7535475

[63] Jones JW, Kayagaki N, Broz P, Henry T, Newton K, O’Rourke K, et al Absent in melanoma 2 is required for innate immune recognition of Francisella tularensis. Proc Natl Acad Sci. 2010;107: 9771–9776. 10.1073/pnas.1003738107 20457908 PMC2906881

[64] Lara-Tejero M, Sutterwala FS, Ogura Y, Grant EP, Bertin J, Coyle AJ, et al Role of the caspase-1 inflammasome in *Salmonella typhimurium* pathogenesis. J Exp Med. 2006;203: 1407–1412. 10.1084/jem.20060206 16717117 PMC2118315

[65] Sutterwala FS, Ogura Y, Szczepanik M, Lara-Tejero M, Lichtenberger GS, Grant EP, et al Critical role for NALP3/CIAS1/cryopyrin in innate and adaptive immunity through its regulation of caspase-1. Immunity. 2006;24: 317–327. 10.1016/j.immuni.2006.02.004 16546100

[66] Marim FM, Silveira TN, Lima DS, Zamboni DS. A Method for Generation of Bone Marrow-Derived Macrophages from Cryopreserved Mouse Bone Marrow Cells. PLoS One. 2010;5: 1–8. 10.1371/journal.pone.0015263 21179419 PMC3003694

[67] Karlen Y, McNair A, Perseguers S, Mazza C, Mermod N. Statistical significance of quantitative PCR. BMC Bioinformatics. 2007;8: 1–16. 10.1186/1471-2105-8-131 17445280 PMC1868764

[68] Pabinger S, Rödiger S, Kriegner A, Vierlinger K, Weinhäusel A. A survey of tools for the analysis of quantitative PCR (qPCR) data. Biomol Detect Quantif. Elsevier GmbH; 2014;1: 23–33. 10.1016/j.bdq.2014.08.002 27920994 PMC5129434

[69] Fumagalli MJ, de Souza WM, Espósito DLA, Silva A, Romeiro MF, Martinez EZ, et al Enzyme-linked immunosorbent assay using recombinant envelope protein 2 antigen for diagnosis of Chikungunya virus. Virol J. Virology Journal; 2018;15: 112 10.1186/s12985-018-1028-1 30041676 PMC6056935

[70] Bancroft JD, Stevens A. Theory and Practice of Histological Techniques. New York, NY: Churchill Livingstone; 1996.

[71] Morrison TE, Oko L, Montgomery SA, Whitmore AC, Lotstein AR, Gunn BM, et al A mouse model of chikungunya virus-induced musculoskeletal inflammatory disease: Evidence of arthritis, tenosynovitis, myositis, and persistence. Am J Pathol. Elsevier Inc.; 2011;178: 32–40. 10.1016/j.ajpath.2010.11.018 21224040 PMC3069999

[72] Cunha TM, Verri WA, Vivancos GG, Moreira IF, Reis S, Parada CA, et al An electronic pressure-meter nociception paw test for mice. Brazilian J Med Biol Res = Rev Bras Pesqui medicas e Biol. 2004;37: 401–7. 10.1103/PhysRevB.54.9449 15060710

[73] Sant’Anna MB, Kusuda R, Bozzo TA, Bassi GS, Alves-Filho JC, Cunha FQ, et al Medial plantar nerve ligation as a novel model of neuropathic pain in mice: Pharmacological and molecular characterization. Sci Rep. Nature Publishing Group; 2016;6: 1–13. 10.1038/srep26955 27230787 PMC4882539

