# Supplementary figures and images for: The NLRP3 inflammasome is involved with the pathogenesis of Mayaro virus
Source: PLoS Pathog. 2019 Sep 3;15(9):e1007934. doi: 10.1371/journal.ppat.1007934 (PMC6743794; doi:10.1371/journal.ppat.1007934)

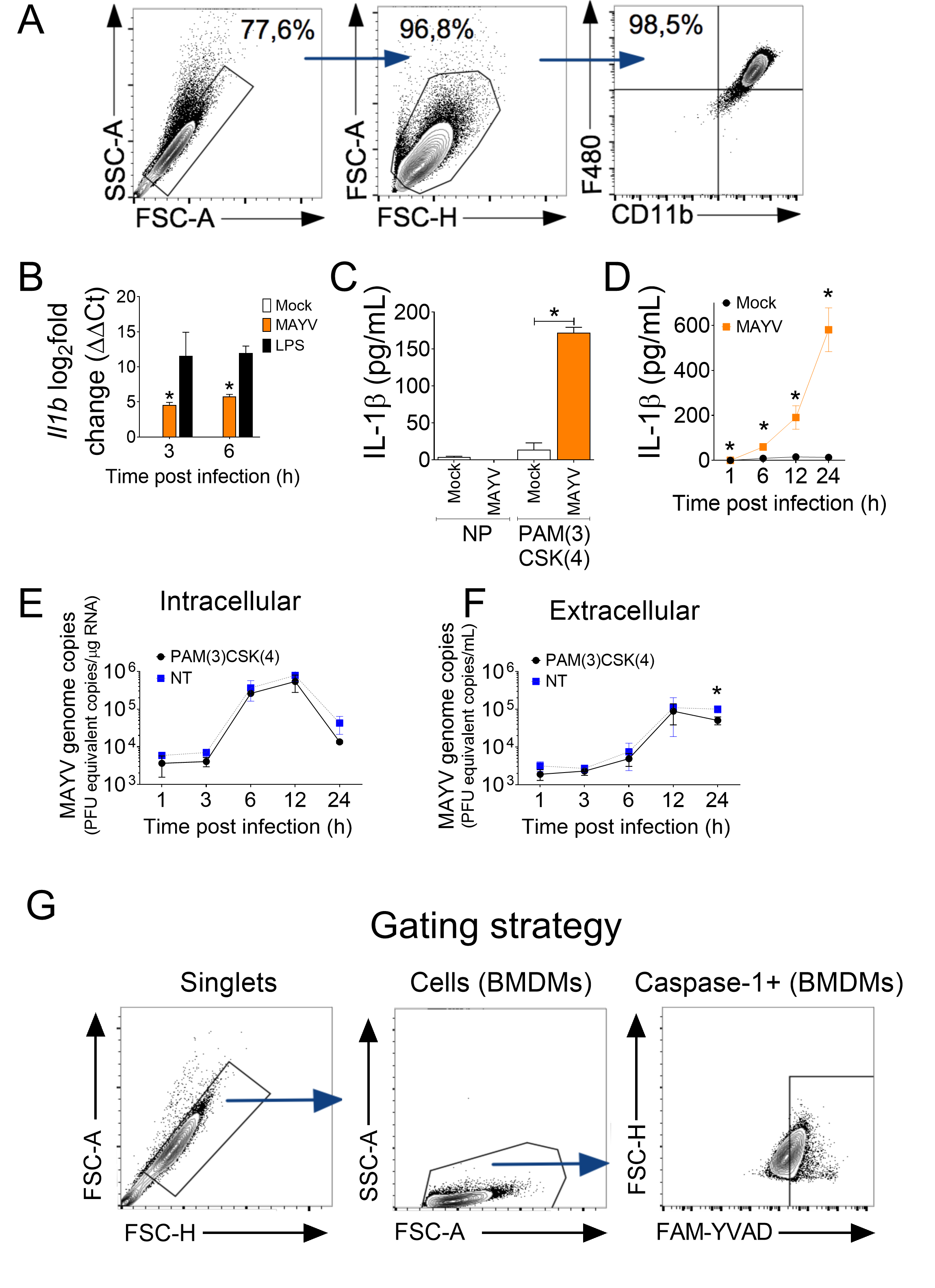

Supplement: S1 Fig — (A) FACS analysis was employed to demonstrate the percentage of mature BMDMs in our culture at 6 days of differentiation. Initially, cells were gated to exclude doublets (FSC-A, FSC-W), and then to exclude debris (SSC-A, FSC-A). The double-positive population represents mature macrophages (CD11b+F4/80+). (B) WT BMDMs were infected with MAYV at a MOI of 5, or treated with RPMI medium (NI), or condoned media (mock), or ultrapure LPS (500 ng/ml) as negative and positive controls, respectively. After 3 or 6 hours of infection, cells were lysed and RNA was extracted for qPCR analysis of Il1b. Macrophages were primed with PAM(3)CSK(4) (C,D) or left non-primed (NP) (C) for 4 hours and infected with MAYV at a MOI of 5. After 1–24 (D) or 24 hours (C), levels of IL-1β in cell-free supernatants were quantified by ELISA. (E,F) Non-treated (NT) and PAM(3)CSK(4)-primed BMDMs were infected with MAYV at a MOI of 0.2, and at the indicated times total RNA was extracted from the cellular extracts and supernatants to evaluate MAYV intercellular or extracellular mRNA levels by qPCR. (G) Gating strategy used in FACS analyses of FLICA assays employing FAM-YVAD. First, cells were gated to exclude doublets (FSC-A/FSC-H), then to exclude other debris (SSC-A/FSC-A). Data are represented as the means ± SD of triplicate samples (A, B) and are representative of at least two independent experiments that yielded similar results. Statistical analysis was performed by student’s t test. Asterisks indicate statistically significant differences between MOCK and MAYV groups. *P < 0.05. (TIF) [file ppat.1007934.s001.tif]

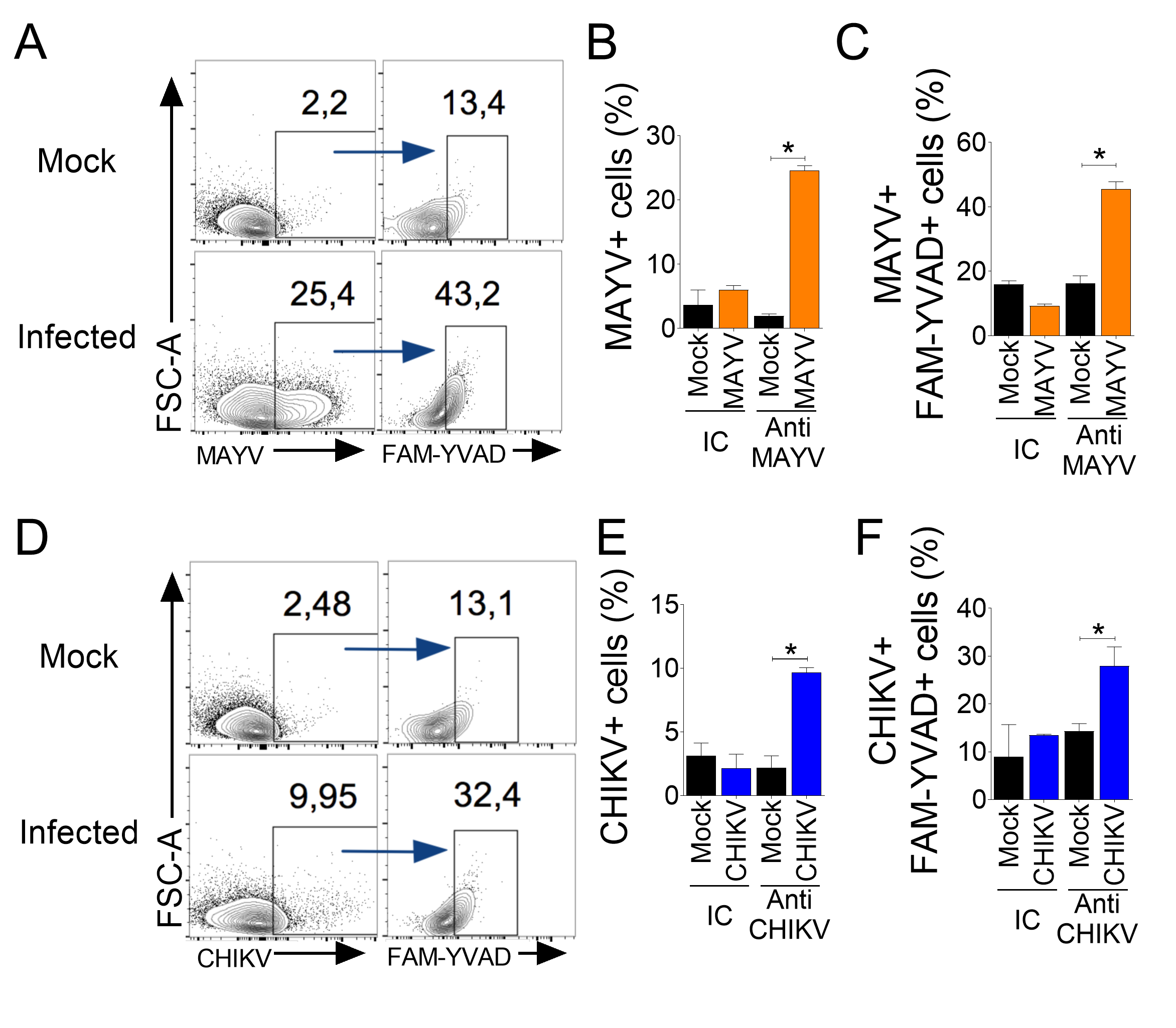

Supplement: S2 Fig — (A) FACS analysis was employed to demonstrate the percentage of BMDMs infected by either MAYV (A-C) or CHIKV (D-F) after 8 hours of infection. The representative contour plots showing the percentages in each plot are shown (A,D), alongside the quantification of MAYV (B) or CHIKV-infected cells (E). MAYV (C) or CHIKV-infected cells (F) were also gated for active caspase-1 (FAM-YVAD). IC: isotype control. Data are represented as the means ± SD of triplicate samples and are representative of two independent experiments that yielded similar results. Statistical analysis was performed by student’s t test. Asterisks indicate statistically significant differences between MOCK and infected groups. *P < 0.05. (TIF) [file ppat.1007934.s002.tif]

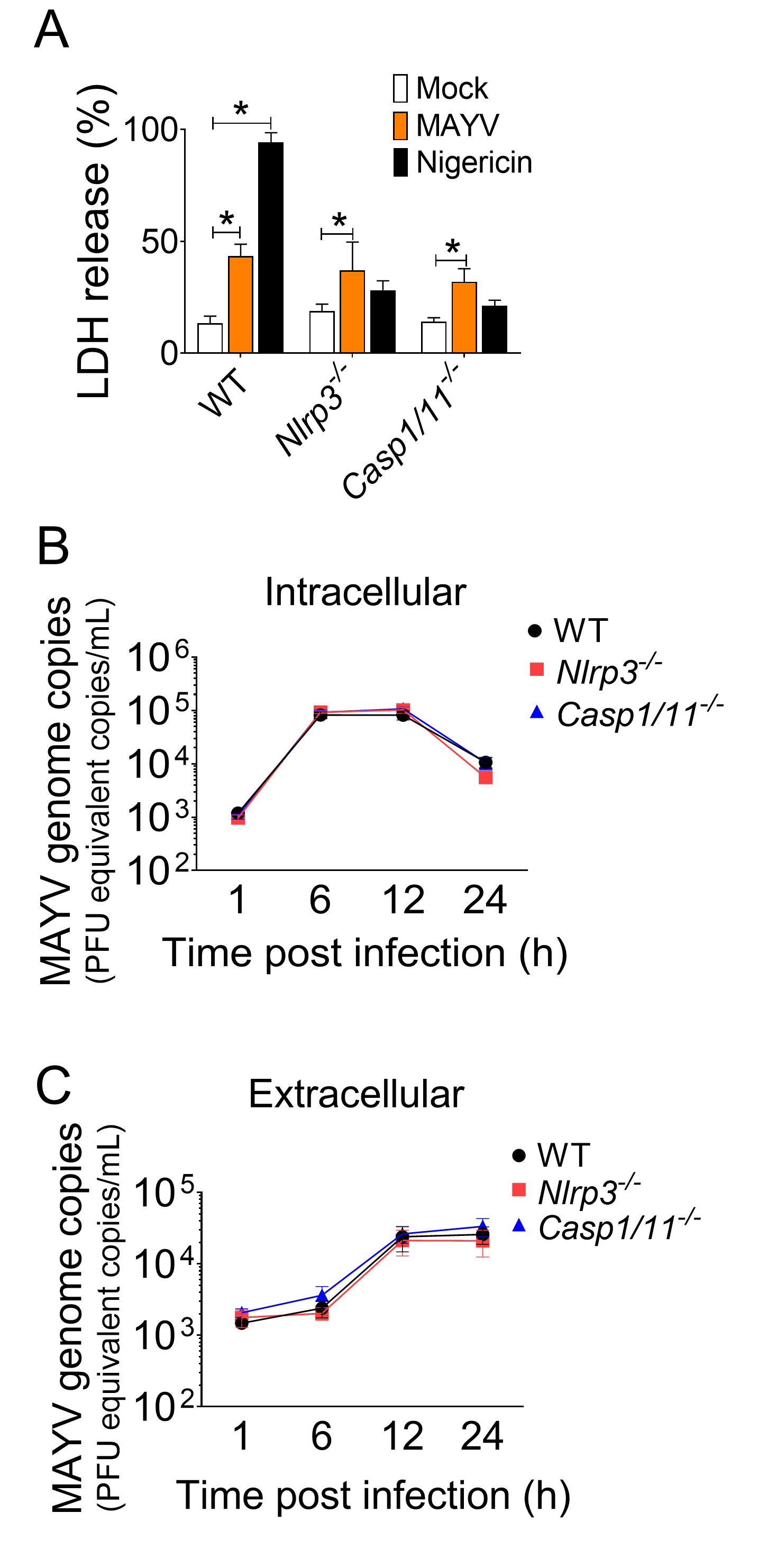

Supplement: S3 Fig — (A) WT, Nlrp3–/–and Casp1/11–/–BMDMs were primed with PAM(3)CSK(4) and then infected with MAYV at a MOI of 5 or mock infected for 24 hours. Cells were also treated with Nigericin (positive control) for 40–60 minutes. Cell-free supernatants were then harvested and LDH release was quantified as previously described. (B) WT, Nlrp3–/–and Casp1/11–/–BMDMs were infected with MAYV at a MOI of 0.2, and at the indicated times total RNA was extracted from the cellular extracts and supernatants to evaluate MAYV intracellular (B) or extracellular (C) mRNA levels by qPCR. Data are represented as the means ± SD of quadruplicate samples and are representative of two independent experiments. Statistical analysis was performed by student’s t test. Asterisks indicate statistically significant differences between groups. *P < 0.05. (TIF) [file ppat.1007934.s003.tif]

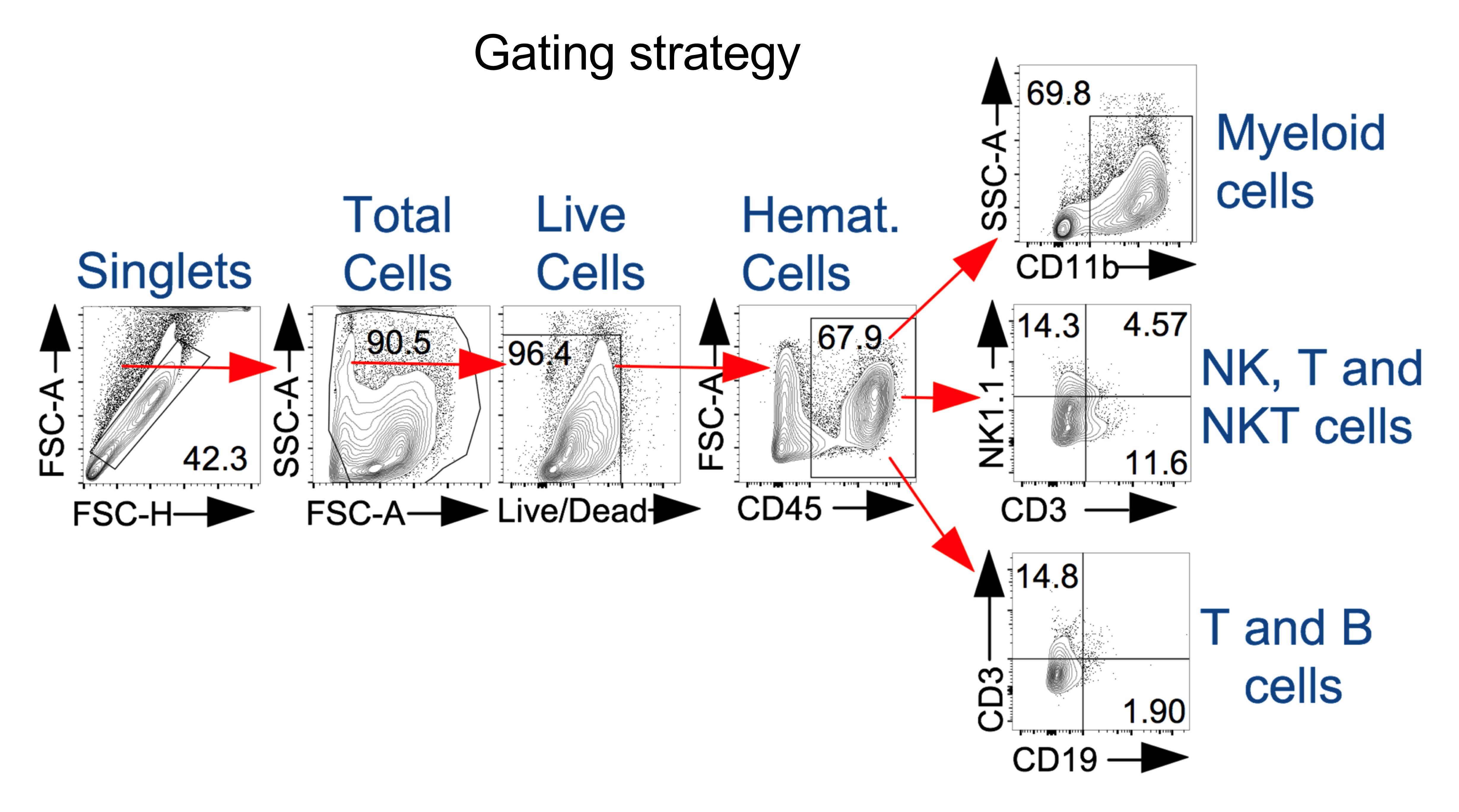

Supplement: S4 Fig — Representative contour plot showing the frequencies of different subsets of hematopoietic cells (CD45+) infiltrating the footpads of MAYV-infected mice. First, cells were gated to exclude doublets (FSC-A/FSC-H), then to exclude other debris (SSC-A/FSC-A). Live cells were selected and hematopoietic cells (CD45+) were gated. Then myeloid (CD11b+), NK (NK1.1+CD3-) and NKT cells (NK1.1+CD3+) and T and B cells (CD3+CD19+) were gated. (TIF) [file ppat.1007934.s004.tif]
